# Activating antiviral immune responses potentiates immune checkpoint inhibition in glioblastoma models

**DOI:** 10.1172/JCI183745

**Published:** 2025-03-17

**Authors:** Deepa Seetharam, Jay Chandar, Christian K. Ramsoomair, Jelisah F. Desgraves, Alexandra Alvarado Medina, Anna Jane Hudson, Ava Amidei, Jesus R. Castro, Vaidya Govindarajan, Sarah Wang, Yong Zhang, Adam M. Sonabend, Mynor J. Mendez Valdez, Dragan Maric, Vasundara Govindarajan, Sarah R. Rivas, Victor M. Lu, Ritika Tiwari, Nima Sharifi, Emmanuel Thomas, Marcus Alexander, Catherine DeMarino, Kory Johnson, Macarena I. De La Fuente, Ruham Alshiekh Nasany, Teresa Maria Rosaria Noviello, Michael E. Ivan, Ricardo J. Komotar, Antonio Iavarone, Avindra Nath, John Heiss, Michele Ceccarelli, Katherine B. Chiappinelli, Maria E. Figueroa, Defne Bayik, Ashish H. Shah

**Affiliations:** 1 Department of Neurosurgery and; 2 Sylvester Comprehensive Cancer Center, University of Miami, Miller School of Medicine, Miami, Florida, USA.; 3 National Institute of Neurological Disorders and Stroke, NIH, Bethesda, Maryland, USA.; 4 Department of Neurological Surgery and; 5 Northwestern Medicine Malnati Brain Tumor Institute of the Lurie Comprehensive Cancer Center, Feinberg School of Medicine, Northwestern University, Chicago, Illinois, USA.; 6 Desai Sethi Urology Institute University of Miami, Miller School of Medicine, Miami, Florida, USA.; 7 Department of Neurology, University of Miami, Miami, Florida, USA.; 8 Biostatistics and Bioinformatics Shared Resource of the Sylvester Comprehensive Cancer Center, University of Miami, Miller School of Medicine, Miami, Florida, USA.; 9 Department of Microbiology, Immunology, and Tropical Medicine, The George Washington University, Washington DC, USA.; 10 Department of Biochemistry and Molecular Biology and; 11 Department of Molecular and Cellular Pharmacology, University of Miami, Miller School of Medicine, Miami, Florida, USA.

**Keywords:** Oncology, Virology, Brain cancer, Cancer immunotherapy, Epigenetics

## Abstract

Viral mimicry refers to the activation of innate antiviral immune responses due to the induction of endogenous retroelements (REs). Viral mimicry augments antitumor immune responses and sensitizes solid tumors to immunotherapy. Here, we found that targeting what we believe to be a novel, master epigenetic regulator, Zinc Finger Protein 638 (ZNF638), induces viral mimicry in glioblastoma (GBM) preclinical models and potentiates immune checkpoint inhibition (ICI). ZNF638 recruits the HUSH complex, which precipitates repressive H3K9me3 marks on endogenous REs. In GBM, ZNF638 is associated with marked locoregional immunosuppressive transcriptional signatures, reduced endogenous RE expression, and poor immune cell infiltration. Targeting ZNF638 decreased H3K9 trimethylation, increased REs, and activated intracellular dsRNA signaling cascades. Furthermore, ZNF638 knockdown upregulated antiviral immune programs and significantly increased PD-L1 immune checkpoint expression in diverse GBM models. Importantly, targeting ZNF638 sensitized mice to ICI in syngeneic murine orthotopic models through innate IFN signaling. This response was recapitulated in recurrent GBM (rGBM) samples with radiographic responses to checkpoint inhibition with widely increased expression of dsRNA, PD-L1, and perivascular CD8 cell infiltration, suggesting that dsRNA signaling may mediate response to immunotherapy. Finally, low ZNF638 expression was a biomarker of clinical response to ICI and improved survival in patients with rGBM and patients with melanoma. Our findings suggest that ZNF638 could serve as a target to potentiate immunotherapy in gliomas.

## Abstract 2

Antiviral immune responses induced by targeting epigenetic mediators of endogenous retroelements potentiates response to immune checkpoint inihbition in GBM.

## Graphical abstract

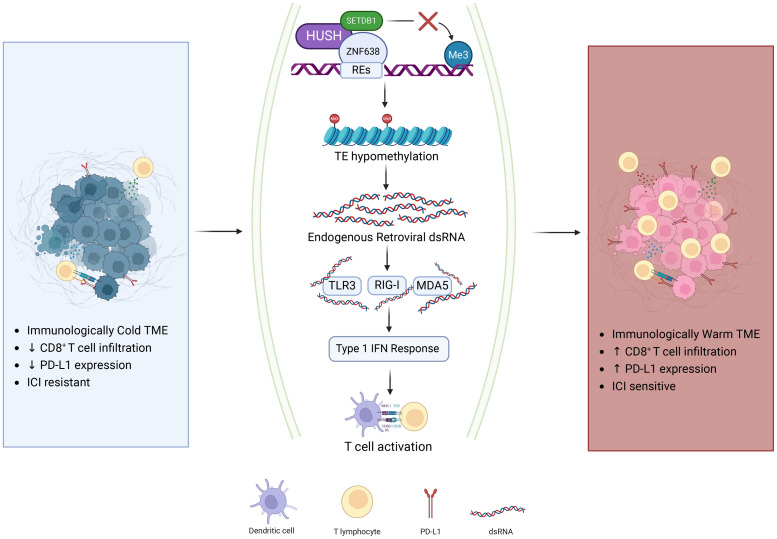

## Introduction

Glioblastoma (GBM), the most common primary brain tumor in adults, has poor median survival rates that have minimally changed over the last 20 years (1). Due to failures in the current standard-of-care treatment regimens, Immune Checkpoint Inhibition (ICI) has been proposed for high-grade gliomas given their success in other solid tumors (2). However, clinical trials using ICI for gliomas have largely failed due to (a) poor tumor antigen presentation, (b) scant intratumoral lymphocyte infiltration, (c) reduced immune checkpoint presentation, (d) poor solid tumor penetration of the immunotherapy, and (e) immune silencing via myeloid-derived suppressor cells/microglia (3, 4). Therefore, strategies to overcome the constitutive immunosuppressive tumor microenvironment of GBM remain vital to improving outcomes for immunotherapy for GBM.

Recently, viral mimicry has been proposed as a strategy to overcome the immunosuppressive tumor microenvironment by activating antiviral antitumor immune responses (5). Viral mimicry refers to an activated antiviral cellular state that is triggered by the epigenetic activation of endogenous nucleic acids, often from retrotransposable elements (cytosolic dsRNA and DNA) (6, 7). Forming more than 40% of our human genome, retrotransposons such as Human Endogenous Retroviruses (HERVs) or LINEs (Long Interspersed Nuclear Elements) are normally silenced by epigenetic modifications such as DNA hypermethylation, chromatin remodeling, and histone modifications. However, epigenetic dysregulation, such as the global DNA/histone demethylation found in GBM, facilitates reactivation of these viral-like sequences, inducing endogenous IFN responses mediated by innate dsRNA sensing pathways (RIG-I and MDA5) (5, 6). Viral mimicry has been used to potentiate tumor cytotoxicity and induce preclinical responses to immunotherapy in a variety of cancers, including colorectal cancer, melanoma, lymphoma, ovarian, and renal cell carcinoma (8–11). Since ICI trials in GBM have largely failed, investigating the role of epigenetic reprogramming and associated viral mimicry–induced immune responses presents an opportunity to enhance the efficacy of ICI.

**Figure 1:**
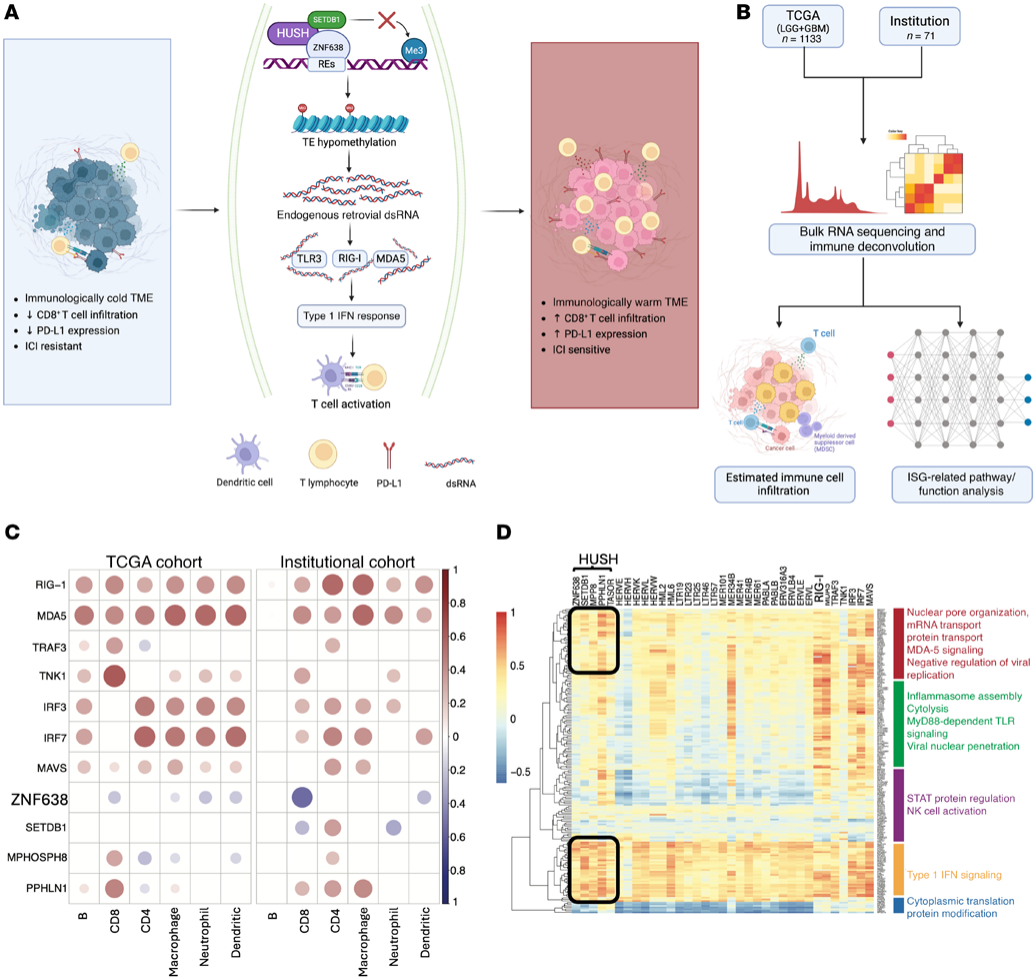
The retroviral silencing complex mediates the suppression of immunogenic RNA species in gliomas. (**A**) ZNF638 acts as the master regulator of a retroviral silencing complex to silence retroelement expression via H3K9 trimethylation. Removal of HUSH-mediated repressive marks enhances antiviral immune responses through innate dsRNA signaling. Made with BioRender. (**B**) Bulk RNA-seq data from TCGA GBM (*n* = 617) and LGG (*n* = 516) cohorts and our institutional cohort (*n* = 71) were analyzed to conduct immune deconvolution and assess IFN-stimulated gene–related (ISG-related) pathways and functions. Made with BioRender. (**C**) The HUSH complex and ZNF638 transcripts are inversely correlated with CD8 immune cell infiltration (R_TCGA_ = – 0.2017; R_inst_ = – 0.5409) based on data obtained from TCGA GBM (*n* = 617), and LGG (*n* = 516) database as well as from our institutional cohort (*n* = 71). (**D**) Correlation matrix demonstrates enrichment of ISGs with expression of several REs as well as a negative association between the HUSH complex and MDA5 signaling. Additionally, ZNF638 and the HUSH complex are directly correlated with increased inhibition of NK cell activation and Type 1–IFN signaling. Genes assigned to pathways based on the Reactome Pathways database and Gene Ontology analysis. TE, transposable elements.

Histone modifications, specifically H3K9me3, are the predominant epigenetic regulatory mark of retrotransposons. One of the main regulatory mechanisms of H3K9-mediated repression of endogenous retroelements occurs via the human silencing hub (HUSH) complex (12). The HUSH complex is well conserved in mammalian genomes as a host defense mechanism against retroelements. Throughout evolution, the HUSH complex has not only protected against exogenous retroviruses (Human Immunodeficiency Virus, Murine Leukemia Virus), but also against retrotransposition of endogenous retroelements including HERVs and LINE-1 elements (13). The HUSH complex is composed of M-phase phosphoprotein 8 (MPP8), transcription activation suppressor (TASOR), and Periphilin 1 (PPHLN1), which recruits a histone methyltransferase SET domain bifurcated histone lysine methyltransferase 1 (SETDB1) (14). A DNA binding protein ZNF638 has recently been found to be essential in the recruitment of HUSH, which forms the retroviral silencing complex (RSC) to ultimately precipitate H3K9me3 repressive epigenetic marks on unintegrated retroviral DNA (12). When this mechanism is lost in tissues, H3K9me3 repressive marks are removed and cells become susceptible to endogenous retrotransposition, increasing HERV and LINE-1 expression and ultimately antiviral immune responses (12, 13) (Figure 1A). This viral mimicry effect has been demonstrated in other tumors where retroelements increased antitumor immunity and sensitized tumors to ICI (15, 16). Here, we investigated the role of epigenetic reprogramming and associated viral mimicry–induced immune responses to enhance the efficacy of ICI in GBM through an epigenetic regulator, ZNF638.

## Results

### ZNF638 is associated with a unique epigenetic and immunological landscape in GBM.

Given the established role of ZNF638 in epigenetic silencing of retroelements, we sought to understand its association with dsRNA-sensing pathway activation in GBM. (Figure 1A and Supplemental Figure 1, A and B; supplemental material available online with this article; https://doi.org/10.1172/JCI183745DS1). To gain insight into the role of ZNF638 in shaping the epigenetic and immunological characteristics of GBM, we performed immune deconvolution using RNA sequencing from The Cancer Genome Atlas (TCGA) and validated results in an independent institutional cohort of high-grade gliomas (Figure 1B). ZNF638 was significantly negatively correlated with dsRNA sensing pathways (RIG-I, MDA5, TLR3) in GBM and positively associated with members of the HUSH complex and its effectors (SETDB1 and MPHOSPH8) (Supplemental Figure 1C). Leveraging the Tumor Immune Estimation Resource (TIMER) (17), we evaluated the association between the HUSH complex and the RIG-I–sensing pathways with tumor lymphocyte infiltration. ZNF638 was strongly negatively correlated with CD8^+^ T cell infiltration in 2 independent data sets. Conversely, the viral mimicry cascade is also correlated with immune cell infiltration (CD8, CD4, DC, NK, Macrophages). (Figure 1C) To recapitulate these findings, we demonstrated that ZNF638 exhibited a positive correlation with the HUSH components and a negative correlation with dsRNA-sensing programs in brain tumor tissue based on data derived from the ARCHS4 dataset. These correlations are maintained when visualizing genome wide coexpression (18, 19) (Supplemental Figure 2A).

**Figure 2:**
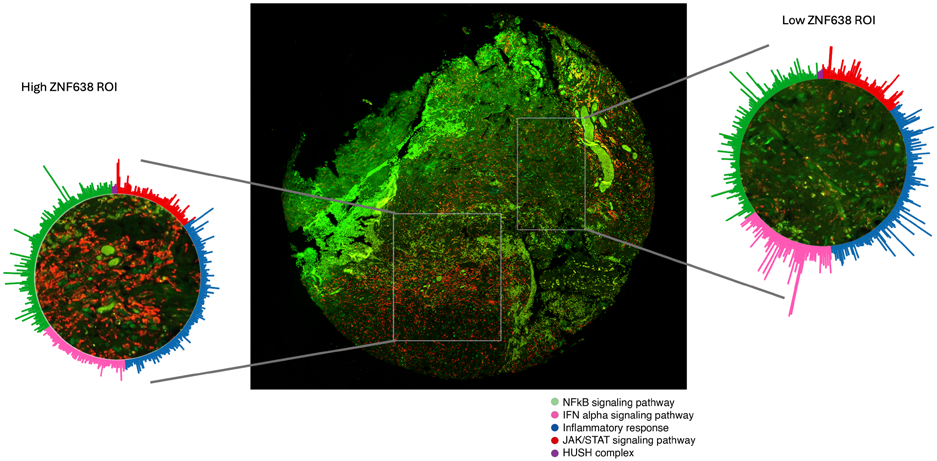
dsRNA-sensing and IFN-signaling pathways are transcriptionally upregulated in high-ZNF638 regions. Circular visualization of gene expression in regions of high and low ZNF638 from a sample from a patient diagnosed with IDH WT recurrent GBM. Utilizing the NanoString GeoMx Digital Spatial Profiler, gene expression involved for the NFkB signaling pathway (*n*_genes_ = 200), IFN-α signaling pathway (*n*_genes_ = 97), inflammatory response (*n*_genes_ = 200), JAK/STAT signaling pathway (*n*_genes_ = 87), and the HUSH complex (*n*_genes_ = 200), are shown. All gene sets obtained from the molecular signatures database (MSigBr). Central image shows the full tumor sample from the patient. Red, CD45; Green, Olig2.

To understand the landscape of endogenous retroviruses in GBM and their association with the HUSH complex, we utilized a custom bioinformatic pipeline using Telescope, a computational software tool that estimates transposable element expression (20). This comprehensive analysis included the expression of 48 HERV families and the components of the HUSH/ZNF638 complex. Our findings indicated that the expression of HUSH effector proteins, PPHLN1, and TASOR exhibited widespread negative associations with several HERV families (Supplemental Figure 2B). Furthermore, using hierarchical clustering from human GBM specimens, we discovered that ZNF638 expression was associated with the downregulation of MDA5 signaling, while multiple HERV loci were enriched in the Type 1–IFN cellular signaling programs (Figure 1D). Utilizing spatial transcriptomics, we demonstrated that regions of interest with low expression of ZNF638 demonstrated upregulated transcription of components of the *IFNA* and *NFKB* signaling pathways, as annotated by the Molecular Signatures Database (MSigDb) (Figure 2) (21).

**Figure 3:**
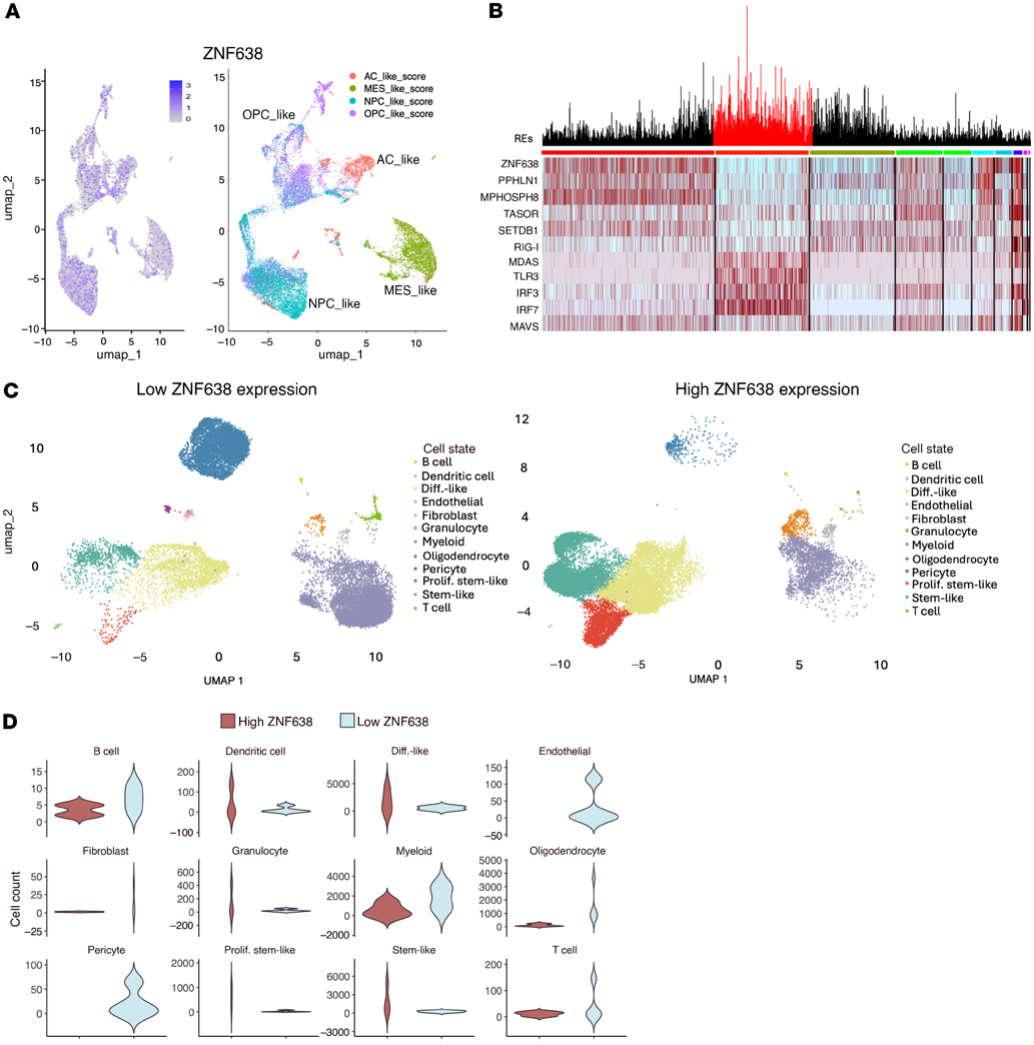
ZNF638 suppresses total RE expression and dsRNA sensing. (**A**) Single-cell RNA-seq and clustered analysis depicting ZNF638 expression and Neftel state classification (*n* = 18,400 cells) (NPC-like, neural-progenitor-like; OPC-like, oligodendrocyte-progenitor-like; AC-like, astrocyte-like; MES-like, mesenchymal-like). (**B**) Heatmap depicting reduced cellular expression of the retroviral silencing complex (ZNF638, SETDB1, PPHLN1, MPHOSPH8, TASOR) is associated with increased total retroelement expression and increased expression of genes involved in the dsRNA sensing pathway (RIG-I, IFIH1, TLR3, IRF3, IRF7, MAVS) (*n* = 18,400 cells, REs = 5,680). (**C**) Low ZNF638 expression is associated with increased lymphocyte expression in individual GBM tumors using unsupervised clustering. UMAPs from tumors with low (*n*_tumors_ = 4, *n*_cells_ = 17,535) and high (*n*_tumors_ = 4, *n*_cells_ = 20,517) ZNF638 expression show heterogenous and distinct enrichment of cell types. (**D**) Violin plots illustrate the expression levels of B cells, dendritic cells, differentiated-like cells, endothelial cells, fibroblasts, granulocytes, myeloid cells, oligodendrocytes, pericytes, proliferative stem-like cells, stem-like cells, and T cells in tumors with low versus high ZNF638 expression, based on unbiased cell type annotation. While a trend toward increased infiltration of T cells, B cells, myeloid cells, and oligodendrocytes is observed, this did not reach statistical significance, likely due to the limited number of samples. Single-cell data for panels **A**–**D** were obtained from the European Genome-Phenome Archive under accession number EGAS00001005300.

To understand the role of ZNF638 and the HUSH complex in GBM cellular states and the tumor environment, we leveraged a single-cell RNA-seq dataset of 11 adult patients with glioma to evaluate the effect of *ZNF638* expression on the expression of HERV families and associated dsRNA-sensing pathway components (22). Retroelements were characterized in this dataset using TE-transcripts to create a custom dataset with a robust representation of the transcriptome and retrotranscriptome in glioma. Malignant cells lacking *ZNF638* expression were significantly enriched in total retroelement expression (both coding and noncoding RNA elements). *ZNF638* expression was associated with distinct cell-state clustering, with significantly lower expression in mesenchymal-like cells. (Figure 3A) Cellular transcription states have been characterized as oligodendrocyte-progenitor-like (OPC, high *PDGFRA*), astrocyte-like (AC, high *EGFR*), mesenchymal-like (MES, *NF1* alteration), and neural-progenitor-like (NPC, high *CDK4*) (23). There is evidence that MES-like tumors demonstrate increased tumor-associated macrophages and cytotoxic T cell enrichment (24). Malignant cells with low *ZNF638* expression demonstrated increased expression of total retroelements and increased expression of dsRNA-sensing pathway regulators (*RIG-I*, *TLR3*, *MAVS*, *MDA5*, *IRF3*, *IRF7*) (Figure 3B). Furthermore, when stratifying individual tumors based on *ZNF638* expression, we identified that low *ZNF638*-expressing tumors were associated with increased tumor-infiltrating lymphocytes (Figure 3C). Differential infiltration of specific lymphocytes and cell types demonstrated a trend of increased infiltration of B cells, Myeloid Cells, T cells, and Oligodendrocytes; however, *P* values were greater-than 0.05 due to the small total number of immune cells (Figure 3D).

**Figure 4:**
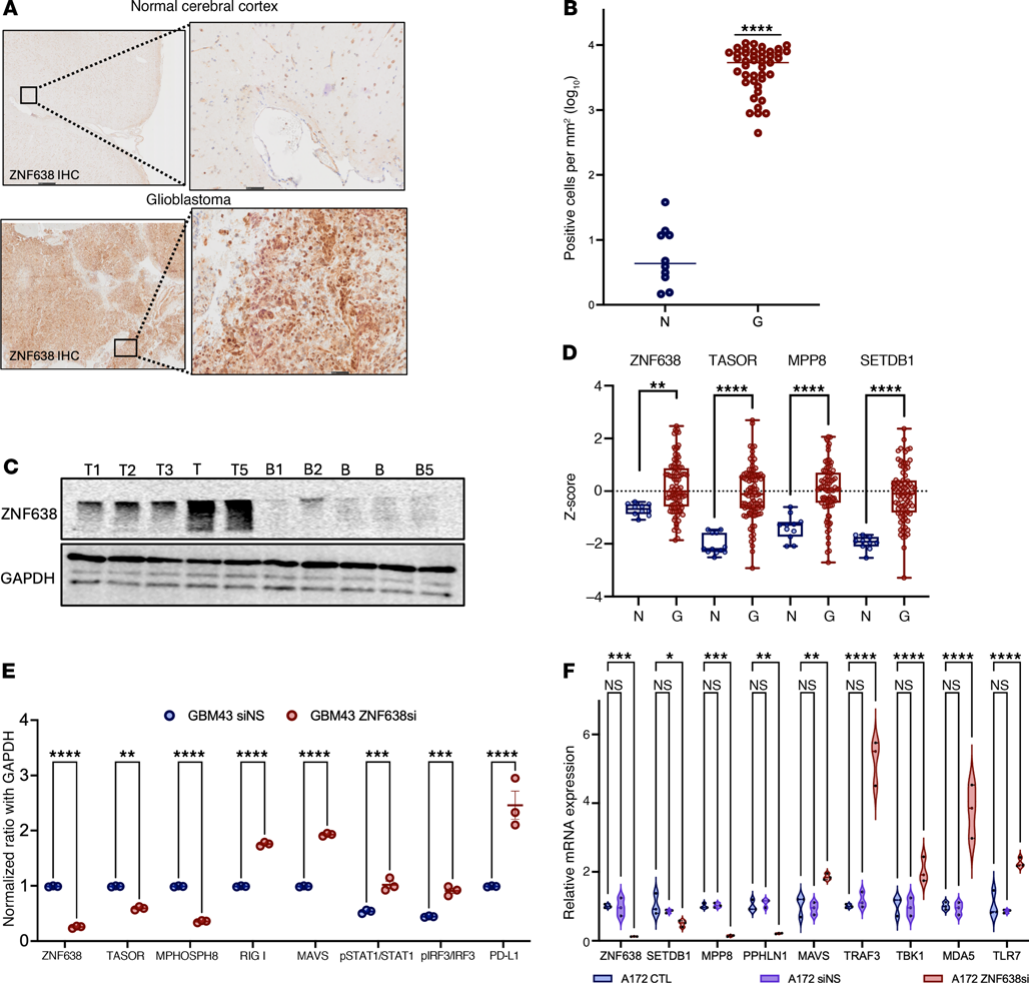
ZNF638 expression is enriched in GBM and induces dsRNA signaling when knocked down. (**A** and **B**) IHC staining and quantification for ZNF638 demonstrates marked overexpression of ZNF638 in GBM tumors compared with matched normal cortex (*n* = 43 versus 10, *P* < 0.0002). Results independently verified in biological replicate from 2 separate patient cohorts. (**C**) Western blot demonstrates that ZNF638 expression is enriched in GBM tumor samples compared with patient-matched adjacent normal brain (*n*_T_ = 5 versus *n*_C_ = 5). (**D**) Proteomic data from the Clinical Proteomic Tumor Analysis Consortium (CPTAC) data portal for GBM (*n* = 12) and normal tissue (*n* = 99) corroborates significant enrichment of ZNF638, TASOR (FAM208A), MPHOSPH8, and SETDB1 in tumor tissue. (**E**) Western blot quantification validates that ZNF638 transient KD by siRNA reduces expression of HUSH via MPP8 and increases expression of RIG-I, MAVS, TBK1, pIRF3, and pSTAT1 in patient-derived GBM43 (1-way ANOVA, performed in technical triplicate, ****P < 0.0001, ***P < 0.001, **P < 0.01). (**F**) Knockdown of ZNF638 by siRNA decreases expression of SETDB1, PPHLN1, and MPP8 as well as increases expression of MAVS, TRAF3, TBK, MDA5, and TLR7 as measured by qPCR in A172 cells (performed in technical triplicate, 1-way ANOVA, ****P < 0.0001, ***P < 0.001, **P < 0.01).

To confirm the clinical relevance of ZNF638 in GBM, we demonstrated that ZNF638 was uniquely enriched in GBM tissue relative to matched normal cerebral cortex via IHC and Western blot (Figure 4, A–C, and Supplemental Tables 1 and 2). Using data from the National Cancer Institute Clinical Proteomic Tumor Analysis Consortium (CPTAC) we further demonstrated that ZNF638, TASOR, MPHOSPH8, SETDB1 proteins were significantly enriched in GBM versus normal brain tissue (Figure 4D).

### ZNF638 knockdown suppresses HUSH expression and induces innate antiviral immune signaling.

Given these findings, we sought to decipher the role of ZNF638 in regulating antiviral immune signaling via epigenetic reprogramming of the retroviral silencing complex (RSC). To assess the role of ZNF638 in mediating the HUSH complex and dsRNA signaling pathway, we transiently downregulated *ZNF638* in diverse GBM cell lines using RNA interference. Knockdown of *ZNF638* resulted in a notable reduction in the expression of the RSC complex, including *SETDB1*, *PPHLN1*, and *MPP8,* as well as an increase in the innate antiviral signaling cascade, including *MAVS*, *TRAF3*, *TBK1*, *pIRF3*, and *TLR7* (Figure 4, E and F, and Supplemental Figure 3, A and B) (25).

**Figure 5:**
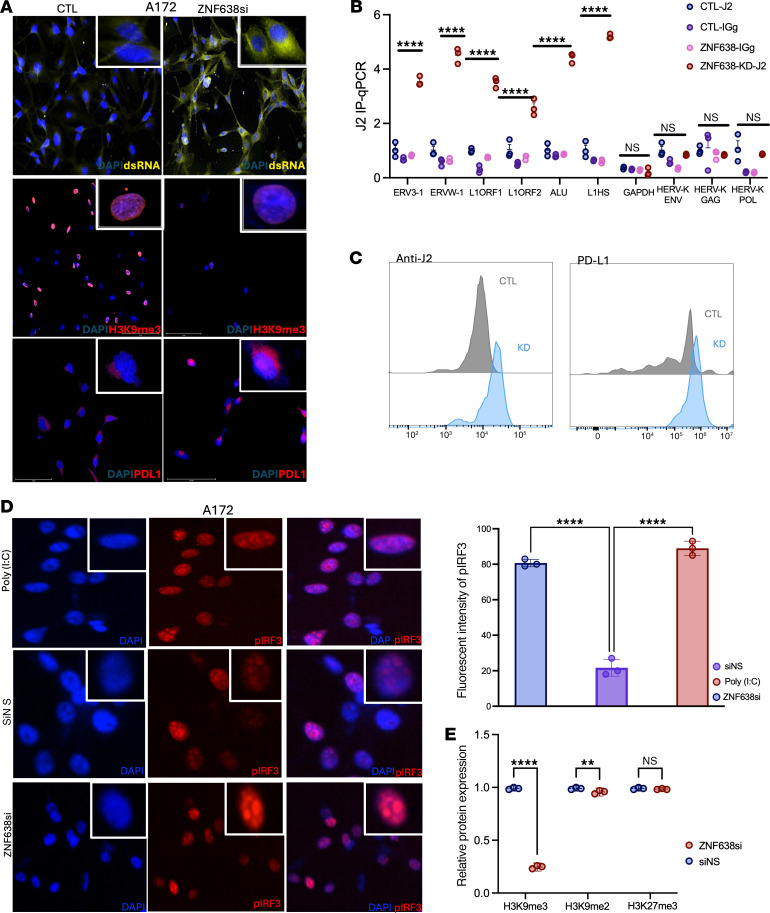
ZNF638 KD increases dsRNA expression secondary to loss of H3K9me3 signature. (**A**) ZNF638 knockdown by siRNA in A172 GBM cells demonstrates increased expression of dsRNA (yellow), decreased H3K9me3 (red), and increased expression of PD-L1 (red), as evidenced by quantitative immunofluorescence. Nuclei are stained blue with DAPI. (**B**) RNA-immunoprecipitation (J2 antibody) demonstrates increased pulldown of RE dsRNA with ZNF638 KD in A172 cells (performed in technical triplicate, 1-way ANOVA, ****P < 0.0001). (**C**) Flow cytometry demonstrates increased expression of dsRNA (anti-J2) and PD-L1 with ZNF638 KD. (**D**) ZNF638 knockdown elicits antiviral immune signaling via increased expression of pIRF3 (red) via quantitative immunofluorescence. Poly I:C (40 ng/mL) represents a positive control for pIRF3 signaling. Nuclei are stained blue with DAPI. (1-way ANOVA, ****P < 0.0001). (**E**) Knockdown of ZNF638 with siRNA results in loss of H3K9me3 in A172 cells based on Western blot (performed in technical triplicate, ****P < 0.0001, *P < 0.05). ZNF638-KD does not change H3K27 trimethylation.

To understand the molecular underpinnings of the viral mimicry cascade, we sought to understand the relationship between ZNF638, H3K9 trimethylation, and dsRNA expression. Using dsRNA immunoprecipitation (J2 antibody, we demonstrated that ZNF638 knockdown significantly increased dsRNA expression in GBM A172 cells (Figure 5, A–C). Specifically, we found broadly increased expression of retroelements including LINE-1, Alus, and LTRs, with relatively no change in HERV-K RNA species (Figure 5B). This global increase in dsRNA intermediates was secondary to a reduction in H3K9 trimethylation. To confirm this result, we validated these findings using quantitative immunofluorescence and flow cytometry. Importantly, this viral mimicry activation was associated with an increase in immune checkpoint presentation (PD-L1) in GBM cells, as demonstrated by immunofluorescence, Western blot, and flow cytometry (Figure 5, A and C). Additionally, ZNF638 knockdown induced downstream antiviral dsRNA signaling, as evidenced by phospho-IFN regulatory factor 3 (pIRF3) expression on immunofluorescence (Figure 5D). Using coimmunoprecipitation, we demonstrated that knockdown of ZNF638 resulted in significantly decreased levels of TASOR, MPP8, and SETDB1 in GBM cells, suggesting that ZNF638 was critical in maintaining the integrity of the HUSH complex and mediating H3K9me3 repressive histone marks (Figure 5E and Supplemental Figure 3, C and D). Additionally, we demonstrated that ZNF638 KD in A172 significantly increased mitochondrial dsRNA levels compared with the control, which may contribute to RIG-I/MAVS activation (Supplemental Figure 3E). In combination, these results signify that ZNF638 KD neutralizes HUSH-mediated H3K9 trimethylation in GBM and results in increased dsRNA signaling to stimulate the innate antiviral immune response. We also demonstrate ZNF638 KD does not affect proliferation or invasion in A172 (Supplemental Figure 4).

**Figure 6:**
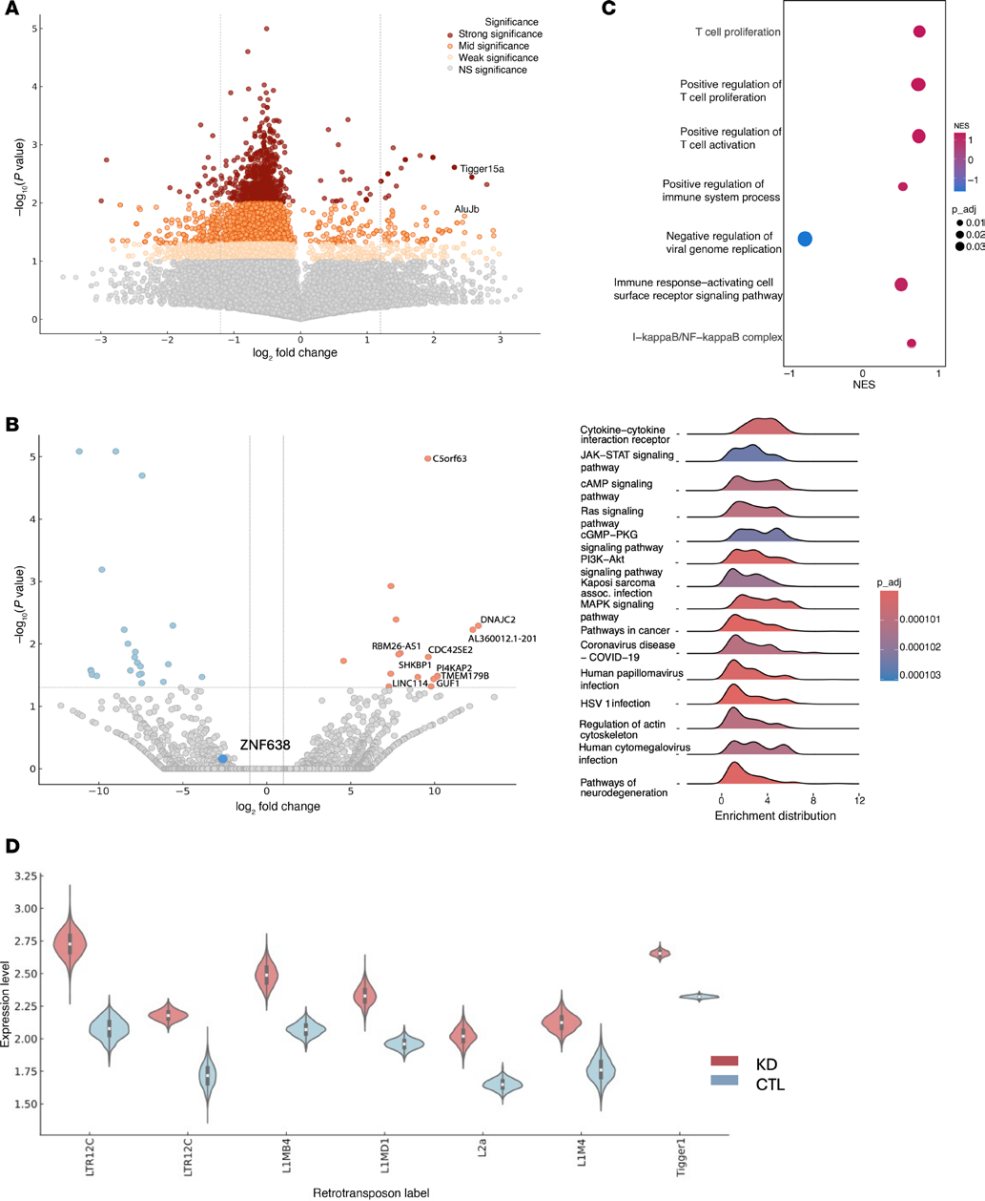
ZNF638 knockdown upregulates retroelement expression and corresponding antiviral and immune programs. (**A**) Volcano plot shows differential analysis of ATAC-seq and demonstrates that ZNF638 KD in a patient-derived GBM cell line results in opening of genomic regions associated with repeat elements (AluJb, Tigger15a) (log_2_fold change_AluJb_ = 2.69 *P*_AluJb_ = 0.038, log_2_fold change_(Tigger16A)_ = 2.57 *P*_Tigger16A_ = 0.036). (**B**) Volcano plot of differentially expressed genes from a patient-derived GBM43 cell line shows upregulation of transcripts related to the innate immune system (TMEM179B, log_2_fold change = 10.1, *P* = 0.033), actin regulation in activated T cells (CDC42SE2, log_2_fold change = 9.61, *P* = 0.016), and transcriptional activation (DNAJC2, log_2_fold change = 12.6, *P* = 0.0051) in ZNF638 KD. (**C**) ZNF638 KD in patient-derived GBM cell line results in upregulation of antiviral and immune pathways and programs. (**D**) ZNF638 KD in patient-derived GBM cell line results in upregulation of several retrotransposons, including LINE, LTR, and Alu elements (*P < 0.05).

To decipher the role of ZNF638 in epigenetic and transcriptomic signatures in patient-derived GBM neurospheres, we utilized a multiomic bioinformatic approach using ATAC-seq and RNA-seq in patient-derived neurospheres (Figure 6, A and B). Using ATAC-seq, we noted that ZNF638 KD resulted in global epigenetic changes with increased open chromatin around select retrotransposons such as Tigger15a and AluJb (Figure 6A). Similarly, ZNF638 inhibition significantly upregulated innate immune and antiviral programs (Figure 6C). Specifically, ZNF638 KD significantly elevated intronic and retroelement transcripts globally with upregulation of specific retrotransposons (LTRs, LINEs, and Tigger1) utilizing a custom bioinformatics pipeline for retrotransposons. (Supplemental Figure 5, Figure 6D, and Supplemental Tables 3 and 4).

### Viral mimicry activates immune checkpoint blockade in GBM.

**Figure 7:**
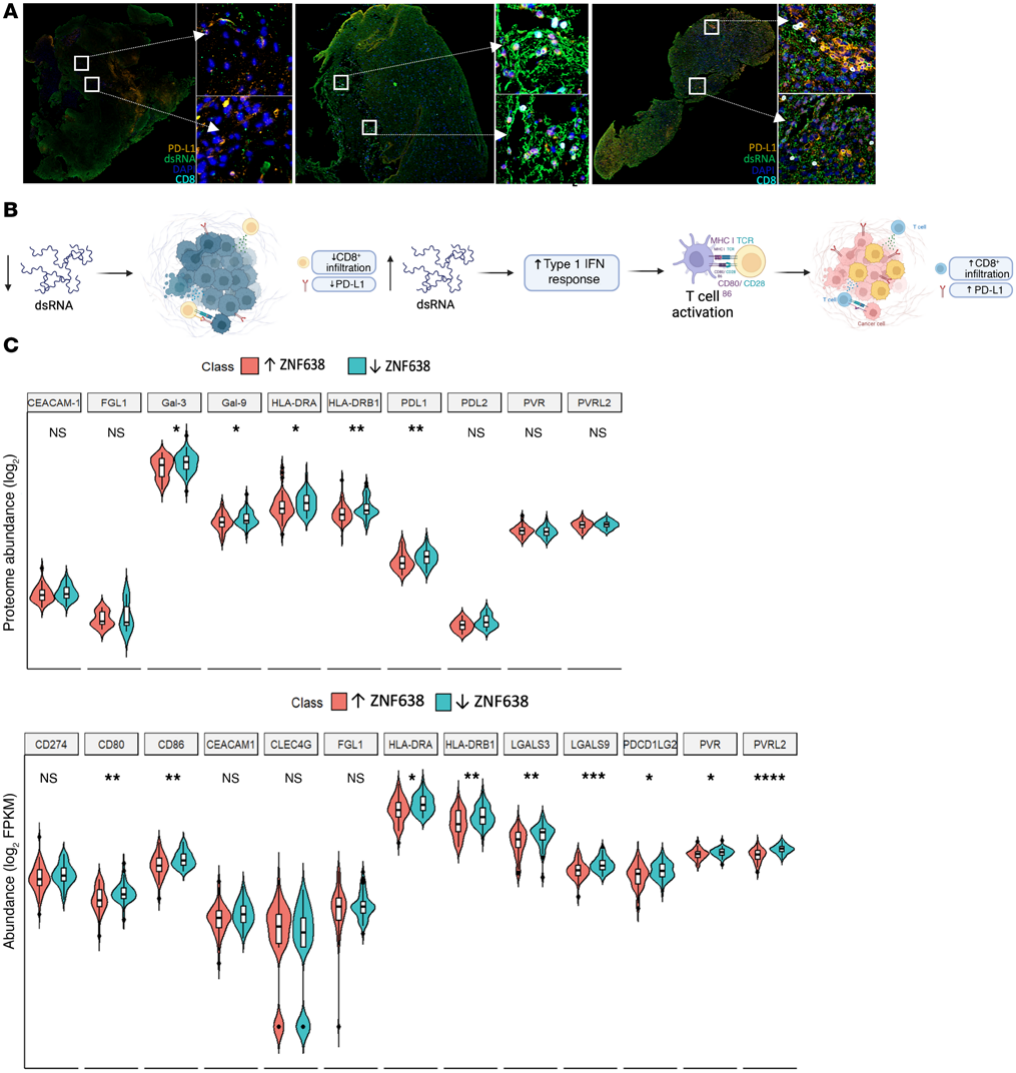
dsRNA expression is associated with increased checkpoint expression and CD8^+^ cell infiltration in high grade gliomas. (**A**) Locoregional dsRNA expression is associated with increased PD-L1 levels and tumor infiltrating lymphocytes in human GBM. Yellow, PD-L1; Green, dsRNA; Blue, DAPI; Cyan, CD8. (**B**) Increased expression of dsRNA stimulates a Type 1–IFN response to induce T cell activation and infiltration and PD-L1 upregulation. Made in BioRender. (**C**) Proteomic and transcriptomic data obtained from the CPTAC data portal validate a negative relationship between expression of ZNF638 and immune checkpoint markers: PD-L1 (*P* < 0.001), HLA-DRA (*P* < 0.01), and HLA-DRB1 (*P* < 0.001) (*n*_low_ = 50 and *n*_high_ = 50 in each group).

To gain insight into the clinical impact of dsRNA expression on local tumor microenvironment, we employed multiplex immunofluorescence of naive GBM, demonstrating an association between baseline dsRNA expression and PD-L1 expression and CD8^+^ T cell infiltration in patients treated with immunotherapy after resection (Figure 7, A and B, and Supplemental Table 5).

As previously demonstrated in lymphoma, melanoma, and colorectal cancer, activation of viral mimicry immune responses increases immune checkpoint presentation in solid tumors (15, 16). Similarly, we discovered that ZNF638 KD significantly increased PD-L1 expression in multiple GBM cell lines (A172 and U87) via Western blot, immunofluorescence, and flow cytometry. This suggests that dsRNA-sensing antiviral programs may potentiate immunotherapy in GBM (26) (Figure 5, A and C, and Supplemental Figure 3B). To corroborate the translational potential of our findings, we established stable shRNA knockdown of ZNF638 in murine GBM cell lines that have been previously validated to recapitulate the intrinsic immunosuppressive characteristics of GBM and its tumor microenvironment with poor basal checkpoint presentation. Importantly, ZNF638 KD in SB28 resulted in a substantial increase in expression of TLR3 and PD-L1 expression (Supplemental Figure 6). This relationship was corroborated in patient samples from the CPTAC data portal and TCGA RNA-seq datasets; high PD-L1 expression was significantly increased in patients with low ZNF638 expression (Figure 7C).

**Figure 8:**
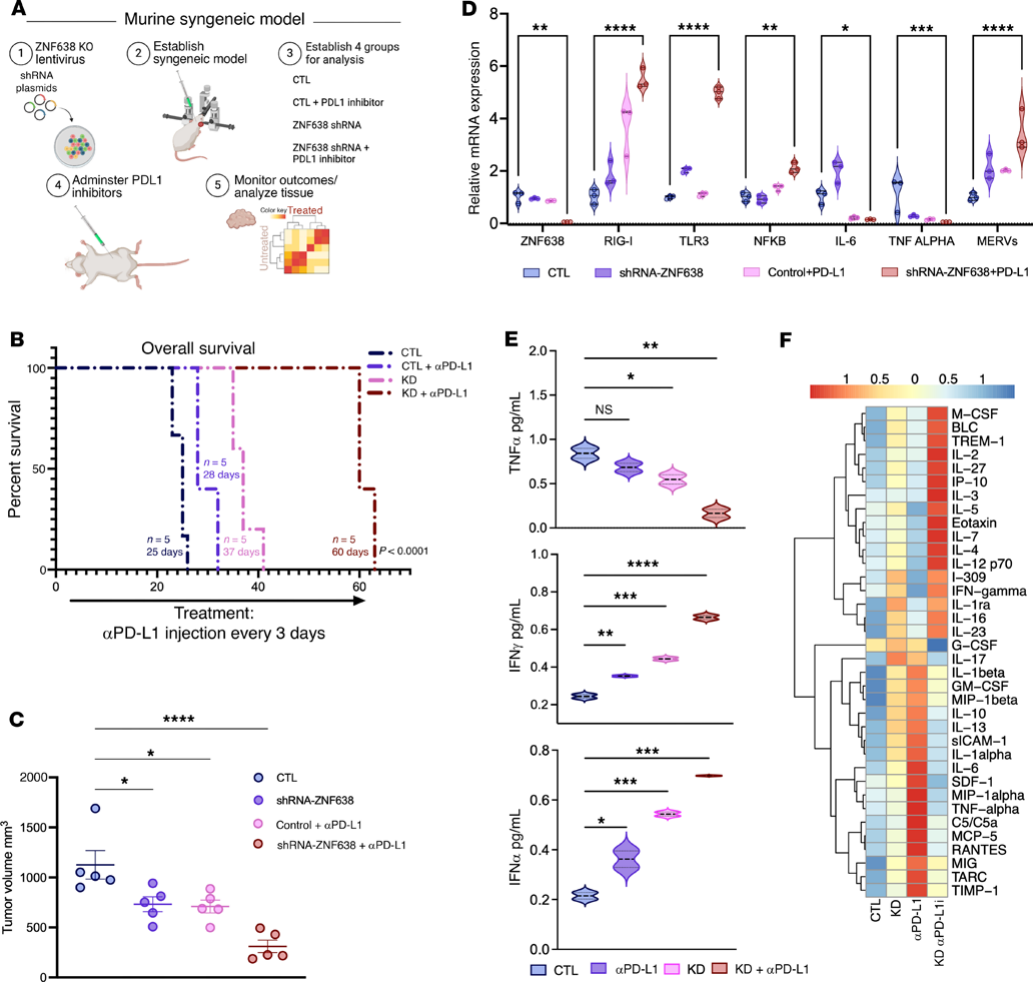
ZNF638 knockdown potentiates ICI response in vivo. (**A**) Diagram of in vivo study design with syngeneic murine GBM model. Made in BioRender. (**B**) Syngeneic GBM murine model with ZNF638 knockdown and PD-L1 inhibition demonstrates significantly improved survival relative to other treatment and control groups (*n* = 5 per group, *P* < 0.01). Multiple animals died on the same day in each group making it appear as if there are fewer animals than were included in each group. (**C**) ZNF638 knockdown and PD-L1 inhibition significantly reduces tumor volume relative to all other groups (1-way ANOVA, ****P < 0.00001, ***P < 0.0001, **P < 0.001, *P < 0.01). (**D**) ZNF638 knockdown + α-PD-L1 shows decreased expression of ZNF638 and increased expression of RIG-I, TLR3, NFK-β, and MERV (RLTR6) transcripts as measured by qPCR. Additionally, there is significant downregulation of IL-6, TNF-α (performed in biological triplicate, 1-way ANOVA, ****P < 0.00001, ***P < 0.0001, **P < 0.001). (**E**) ZNF638 knockdown with PD-L1 inhibition significantly increased expression of IFN-α and IFN-γ, as well as decreased expression of TNF-α (performed in biological triplicate, 1-way ANOVA, ****P < 0.0001, ***P < 0.001, **P < 0.01). (**F**) Proteomic cytokine profiler array with hierarchical clustering depicts distinct cytokine profiles between all treatment/control groups with the greatest difference between CTL+ α-PD-L1 mice and ZNF638 KD + α-PD-L1 mice (1-way ANOVA, *P < 0.05).

To further investigate the targetability of ZNF638 in sensitizing clinical responses to ICIs, we developed an immunocompetent syngeneic orthotopic model employing the SB28 cell line in C57BL/6J mice exposed to checkpoint immunotherapy (Figure 8A). ZNF638-KD mice treated with i.p. αPD-L1 survived significantly longer than control (CTL), ZNF638-KD alone, and sham vector + αPD-L1 groups (Figure 8B). This survival advantage was associated with tumor volumes 90-fold smaller than other experimental groups (Figure 8C and Supplemental Figure 7A). Importantly, ZNF638 KD reduced intratumoral H3K9me3 and increased murine endogenous retroviral expression (RLTR6-M) (Figure 8D and Supplemental Figure 7A). ZNF638 KD with concomitant ICI treatment significantly altered the GBM microenvironment by increasing inflammatory cytokine expression (IL2, IL7, and IP10, and IFNs) and increasing expression of dsRNA-sensing programs (RIG-I, TLR3) (Figure 8, D–F). Consistent with increased IP-10 levels, a chemotactic cytokine that attracts T-cells, ZNF638-KD + αPD-L1 tumor had enhanced CD8^+^ tumor-infiltrating lymphocytes (TILs) compared with other experimental groups. The ZNF638-KD + αPD-L1 group also had greater Natural Killer (NK) cell infiltration compared with all other groups (Supplemental Figure 7A). Additionally, sera isolated from ZNF638-KD mice treated with ICI also revealed significant systemic elevation of IFN-α and IFN-γ levels with reduction of TNF-α levels (Figure 8F). Using multiplex flow cytometry, we demonstrate that synergistic treatment increased populations of CD8^+^, CD4^+^ tumor infiltrating lymphocytes and a shift from monocytic myeloid-derived suppressor cells (mMDSC) to granulocytic myeloid derived suppressor cells (gMDSC). While all MDSCs exhibit potent immunosuppressive activity in the tumor microenvironment, mMDSCs are known to have higher suppressive activity than gMDSCs (27) (Supplemental Figure 7B).

### Viral mimicry is associated with a clinical response to ICI in GBM.

**Figure 9:**
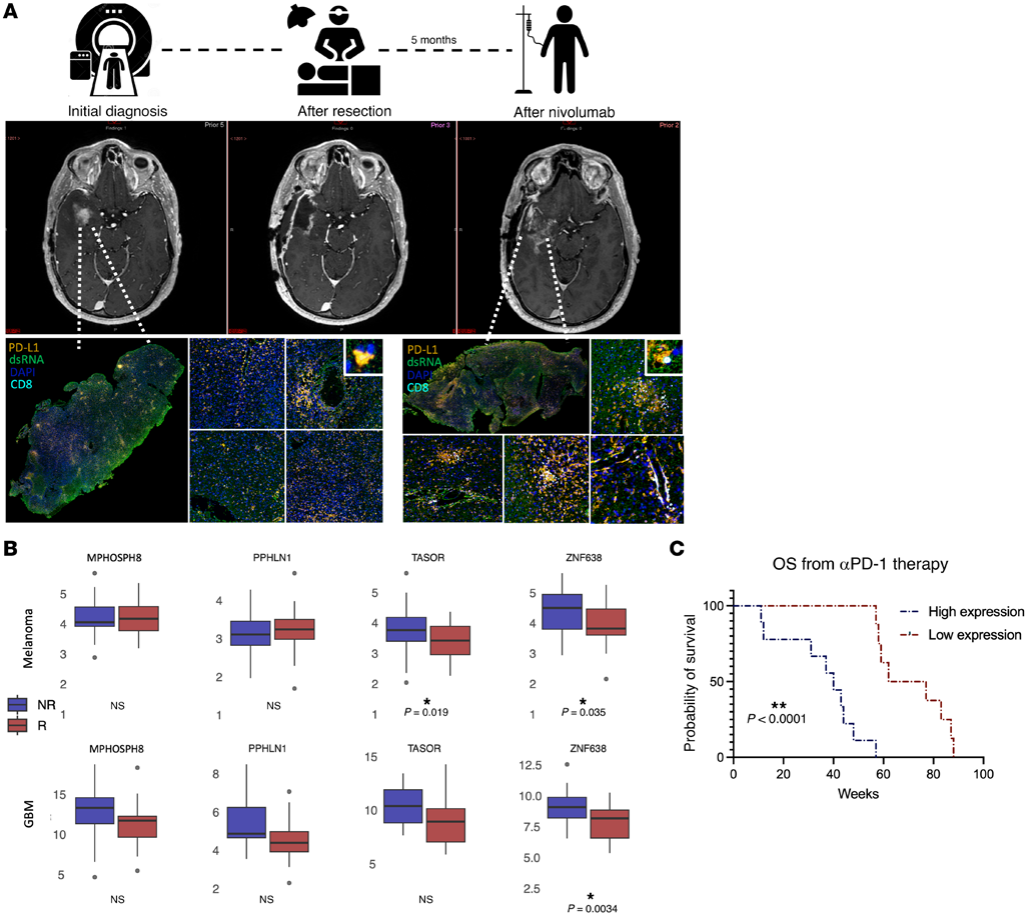
ZNF638 and dsRNA are biomarkers for ICI response in GBM. (**A**) dsRNA expression correlates to increased CD8^+^ T cell infiltration and PD-L1 expression in patients with GBM receiving ICI. 60 year-old male patient with IDH-WT rGBM with temporal contrast enhancing intraaxial tumor concerning for tumor recurrence (MR T1CE) with low baseline CD8 infiltration (cyan) and PD-L1 expression (orange). Postoperative adjuvant ICI (anti-PD-1) resulted in increased enhancement in the tumor cavity 5 months after surgical resection, suggestive of immune pseudoprogression (immune cell infiltration and tumor necrosis). Postresponse multiplex immunofluorescence demonstrates increased dsRNA expression (green) associated with increased CD8 infiltration (cyan) and PD-L1 expression (orange). (**B**) Clinical responders to ICI with rGBM (*R* = 20, *NR* = 18, median = 8.238 versus 9.222 RPKM, *P* = 0.0034) and melanoma (*R* = 34, *NR* = 49, median = 3.855 versus 4.536 RPKM, *P* = 0.035) have markedly lower ZNF638 expression compared with nonresponders to ICI. (**C**) Low ZNF638 expression portends improved survival in recurrent GBM receiving immunotherapy (PD-1 or PD-L1) (Mantel-Cox, *P < 0.01).

To gain insight into the clinical and molecular impact of dsRNA expression on the response to ICIs, we assessed temporal immunological changes in the GBM tumor microenvironment after a clinical response to ICI (nivolumab). In patients with GBM, we noted that immune pseudoresponses were associated with elevated basal dsRNA expression and associated upregulation of PD-L1 expression and CD8^+^ cell infiltration. In ICI-naive tumor specimens, the GBM tumor microenvironment remained relatively subdued, with minimal infiltrating lymphocytes, low dsRNA expression, and low baseline PD-L1 expression. However, after initiation of ICI (anti-PD-1), new enhancement in the tumor cavity revealed histological pseudoprogression with locoregional upregulation of PD-L1 expression in areas with elevated dsRNA expression. This immune pseudoprogression response was characterized by increased infiltration of CD8^+^ T cells and activated microglia (IBA1) in regions enriched with high dsRNA expression (Figure 9A).

Given these findings, we sought to understand the role of ZNF638 in predicting long-term responses to immunotherapy in GBM using genomic and transcriptomic profiling of patients who received ICI (αPD-1, nivolumab, or pembrolizumab). Consistent with our preclinical data, we discovered that patients with ICI-responsive GBM had significantly lower ZNF638 expression than nonresponders and was associated with a significantly improved overall survival (Figure 9, B and C). Importantly, this association was also upheld in other cancer types. In 3 separate cohorts of patients with melanoma who were treated with anti-PD-L1 or anti-CTLA4 therapy, low ZNF638 was significantly predictive of response (Figure 9B). These results suggest that clinical responses to ICI are strongly correlated with innate antiviral immune signatures that could serve as clinical biomarkers for ICI.

## Discussion

GBM is the most common adult primary brain tumor with a dismal prognosis, despite current standard of care (28). Development of novel therapeutics, including immunotherapy for gliomas, has largely failed due to the heterogenous and immunosuppressive tumor microenvironment (29). Therefore, treatment approaches that exploit immunosuppressive molecular marks are highly necessary to improve therapeutic sensitivity for ICI. Gliomas are characterized by a heterogeneous profile of epigenetic dysregulation due to distinct methylation patterns (i.e., IDH1/2 mutations/CpG island methylator phenotype) (30, 31). Previously, we have demonstrated differential expression of retrotransposons in gliomas due to distinct locus-specific epigenetic regulation (32–34). Active transcription of these retroelements (REs) has been demonstrated to improve sensitivity to checkpoint inhibition in colorectal cancer, lymphoma, melanoma, and renal cell carcinoma (7–11, 15, 16). Therefore, capitalizing on the expression of these REs in GBM via targeted epigenetic dysregulation may alter local and systemic immunogenicity and potentiate responses to ICI. Here, we have demonstrated that epigenetic activation of retrotransposon transcription through ZNF638 significantly alters tumor immunogenicity and improves survival via induction of viral mimicry.

The unique epigenetic state of these REs is controlled by the retroviral silencing complex, consisting of ZNF638, SETDB1, and the HUSH complex (MPP8, TASOR, PPHLN1). ZNF638 acts as the master regulator of this complex, responsible for recruiting the HUSH complex and mediating H3K9 trimethylation of retroviral DNA (12). Reexpression of these elements via widespread epigenetic dysregulation (DNMTIs) has been demonstrated to induce dsRNA and dsDNA expression in multiple cancers (15, 35, 36). Other groups have shown that epigenetic reprogramming stimulates the innate antiviral immune response via induction of the RIG-I signaling cascade (37–39). Our results established that targeted alteration of RE epigenetic control through ZNF638 knockdown induces RE-associated dsRNA expression by globally downregulating H3K9me3 and activating the RIG-I/MDA5 pathway in vitro and in syngeneic GBM murine models.

Stimulation of the RIG-I/MDA5 pathway has been established as a critical element for responsiveness to immune checkpoint blockade (anti-CTLA-4 and anti-PD-1) (39). This effect is mediated by Type 1 IFNs and upregulated IFNAR1 (37, 39). ZNF638 knockdown induced activity of RIG-I and its downstream effectors in syngeneic murine glioma models, which resulted in increased PD-L1 expression. Importantly, our results demonstrated that ICI treatment significantly improved survival and reduced overall tumor growth in syngeneic GBM mouse models with ZNF638 knockdown. ZNF638 knockdown altered the GBM tumor microenvironment by enhancing immunogenicity through elevated Type 1–IFN responses and increased CD8^+^ T cell infiltration. This antiviral immune response was conserved in patient-derived GBM neurospheres, which exhibited upregulated immune and antiviral programs with concomitant global loss of genomic repressive marks. In line with these results, a recent study reported that GBM response to immunotherapy, and CD8^+^ T cell recognition, is associated with a MAPK-derived IFN-response phenotype by glioma cells (40).

Previous studies have shown that expression of REs are directly associated with expression of Type I–IFNs via direct binding of specific superfamilies (i.e., HERV-K) to intracellular dsRNA sensors (37, 41). Russ et al. demonstrated that knockdown of MAVS, an associated downstream component of the dsRNA signaling cascade, subsequently reduces expression of Type 1 IFNs and related proinflamamtory cytokines (41). Our transcriptomic analysis of 2 independent glioma datasets further confirmed that ZNF638 expression is negatively correlated with expression of components of the dsRNA signaling pathway. Immune deconvolution of both independent datasets additionally demonstrated that ZNF638 expression is negatively correlated with estimated CD8^+^ and NK cell infiltration. Additionally, transcriptional activity of IFN-stimulated genes and pathways were shown to be inversely correlated with levels of ZNF638 expression. These findings point to the important role of the epigenetic regulators of REs on overall tumor immunogenicity and potential antitumor immune responses. Finally, a comprehensive evaluation of the landscape of retrotransposons demonstrated that multiple components of the retroviral silencing complex (PPHLN1 and TASOR) were widely negatively correlated with most HERV superfamilies. We also demonstrate, with a thorough single-cell transcriptomic analysis, that malignant cells lacking ZNF638 expressed higher levels of total retroelements and were associated with diverse immune cellular profiles.

Overall, our results support a role for ZNF638 as a target to potentiate immune checkpoint inhibition through stimulation of the innate antiviral immune response. This finding was recapitulated longitudinally in the local tumor microenvironment (TME) of GBM responders to ICI. Temporal alterations in the local GBM TME in response to αPD-1 immunotherapy were associated with diffuse dsRNA expression and increases in both PD-L1 expression and CD8^+^ T cell infiltration. More importantly, ZNF638 expression was a biomarker of clinical response to immunotherapy across multiple tumor types, including rGBM and melanoma. Therefore, eliciting dsRNA expression may be a therapeutic modality to potentiate the efficacy of ICI in GBM

Clinical translation of viral mimicry for GBM has been proposed previously through epigenetic reprogramming. Viral mimicry and cytosolic dsRNA expression can be induced through radiation, epigenetic drugs such as DNMTIs or HDACs, and synthetic molecules such as RIG-I agonists (42, 43). However, the lack of specificity and systemic toxicity of epigenetic therapies and synthetic agonists have limited their clinical translation in oncology (44, 45). Synergistic treatments using epigenetic reprogramming and ICI are currently underway in a variety of solid tumors and have shown some promise in early-phase clinical trials (46–49).

Although viral mimicry has been shown to enhance immunotherapy responses in cancer, it is critical to consider the pleiotropic role of REs in the GBM TME. We have previously demonstrated that expression of certain HERV-K families (HML-2, HML-6) are associated with synthesis of full-length retroviral proteins that may contribute to an oncogenic phenotype and tumor stemness (32, 33). In our analysis, targeting ZNF638 did not affect endogenous HERV-K expression, suggesting differential control of older viral dsRNA elements and more recently integrated HERV-K loci. Therefore, we suspect that there remains a balance between antiviral immune programs (overall RE expression) and specific oncogenic viral programs (HERV-K). Further RE-specific methylation profiling may elucidate the specific retroelements associated with viral mimicry immune responses.

Overall, ZNF638 is a biomarker of clinical response to ICI in GBM, suggesting that ZNF638 expression could not only predict clinical responses but may also serve as a target to potentiate immunotherapy across multiple tumor types. It is unknown how ZNF638 may interact with other markers of response to immunotherapy, such as clonal tumor mutational burden, STING activation, DNA replication stress, and MAPK signaling. Future studies are needed to clarify these potential interactions.

Taken together, there may be a clear role for our findings as an adjuvant therapy to both enhance antitumor innate immune responses and potentiate immunotherapy. Overall, these results inform the direct effect of ZNF638 on mediating viral mimicry immune responses in GBM, uncovering an avenue for clinical translation of epigenetic therapies that leverage the retroviral landscape of GBM.

## Methods

### Sex as a biological variable.

Sex was not considered as a biological variable. For clinical samples, both sexes were represented. For our in vivo experiments, only female mice were used. Our findings are expected to be relevant to both sexes.

### Cell culture and transfection.

Neurospheres derived from patient GBM samples (GBM43, IDH WT, 69-year-old male patient) were sourced from the Mayo Clinic Brain Tumor Patient–derived Xenograft National Resource (50). These were cultured in DMEM/F12 with GlutaMAX (Invitrogen), supplemented with 10 ng/mL epidermal growth factor, fibroblast growth factor, B27, N2 (Invitrogen), 1% penicillin-streptomycin, Heparin, and 1% sodium pyruvate (Thermo Fisher Scientific). Established cell lines (A172, U87, and normal human astrocytes) were acquired from ATCC and maintained per the manufacturer’s guidelines in their respective media: A172/U87 in DMEM with 5% FBS and penicillin/streptomycin, the murine SB28 cell line (gifted by Defne Bayik, University of Miami, Miami Florida, USA) in RPMI with 5% FBS and penicillin/streptomycin, and normal human astrocytes in Astrocyte Growth Medium Bullet Kit (Lonza). Neurospheres and established cell lines were dissociated using TrypLE Express (Invitrogen) and cells were not used beyond passage number 20. For ZNF638 knockdown experiments (Thermo Fisher Scientific, AM16708), predesigned siRNAs and scrambled negative control siRNA were utilized. Transfections of siRNA duplexes into A172, U87, and GBM43 cell lines were performed using Lipofectamine RNAiMAX (Thermo Fisher Scientific, 13778150) following the manufacturer’s instructions. Posttransfection, RNA and protein were extracted for downstream analyses including RNA sequencing, ATAC sequencing, Western blotting, and IHC.

### Western blot analysis.

Cells were treated with ZNF638 siRNA and cultured for 72 hours prior to protein collection. Total protein was extracted using RIPA buffer (Sigma-Aldrich) with protease/phosphatase inhibitors (ABCAM). Protein concentrations were determined using Bio-Rad Protein Reagents, following the manufacturer’s protocol. The protein samples were denatured in a mixture of SDSPAGE Reducing Agent (× 10) and RIPA buffer (× 1), separated on a 4%–20% Tris-Glycine SDS-PAGE gel (Bio-Rad), and transferred to nitrocellulose membranes using the iBlot transfer device (Thermo Fisher Scientific). Membranes were blocked with 5% Blotting-Grade blocker in TBS (Bio-Rad) with Tween (Thermo Fisher Scientific), followed by incubation with primary antibodies overnight. After washing with TBS-T, membranes were probed with either anti-rabbit or anti-mouse secondary antibodies (Invitrogen) and visualized using ECL chemiluminescence (Thermo Fisher Scientific). Detailed information about the specific antibodies used is provided in Supplemental Table 6. For histone analysis, histones from A172 cells treated with scramble and ZNF638 siRNA were purified using a histone extraction kit (Active Motif) as per the manufacturer’s instructions. Twenty micrograms of each lysate were separated on a gel and probed for total H3, H3K9me3, and H3K9me2.

### Spatial transcriptomics.

Raw counts from the spatial transcriptomics experiment were exported from Nanostring Geomx into R studio version 4.4.1. The Bioconducter package *GeomxTools* was used for quality control and data filtering. The R package *dplyr* was also used for data manipulation. Regions of interest were sorted into high and low levels of ZNF638 expression. 2 regions from one patient with GBM were analyzed with heterogeneous ZNF638 expression. Expression data was sorted by pathway, using gene sets from the *MSigDB* R package. This was visualized with a radial plot using R package *ggplot2*.

### Analysis of ZNF638 and REs in transcriptional states and cell types.

Single-cell RNA-seq data obtained from Johnson et al. (22). (obtained from the European Genome-Phenome Archive under accession number EGAS00001005300) was annotated using a custom bioinformatics pipeline for retrotransposon counts from TE-transcripts (20) (M. Hammel Laboratory, Cold Spring Harbor Laboratory, Laurel Hollow, New York). This data was analyzed using standardized workflow for object creation, integration, normalization, and visualization in Seurat in RStudio (51, 52). Cells were filtered based on percentage of mitochondrial transcript expression and number of detected features. Filtered data were normalized and scaled using inherent Seurat functions. Cell types and transcriptional states were annotated based on established cell-type and cell-state specific markers (23). A custom script was developed for scoring transcriptional state signatures based on individual cells. Ranked lists were used as input to score each cell for cell type and transcriptional state signatures. These were visualized using DimPlot. The inherent Seurat function FindMarkers was used to calculate log_2_ fold change and adjusted *P* values for unique markers of each cluster. These were used to verify cell type and transcriptional states of clusters labeled by ranked list input. All UMAP plots were generated using Seurat functions RunUMap and DimPlot. All heatmaps and feature plots were visualized using Seurat functions DoHeatmap and FeaturePlot respectively.

### Preparation of cDNA from tumor tissue and cell lines.

RNA was isolated from tumor tissue and cell line samples using the TRIzol extraction protocol. Quantification of RNA was performed using the NanoDrop 2000 (Thermo Fisher Scientific), and the concentration was adjusted to 1000 ng/μL. The RNA was then reverse transcribed using the iScript cDNA Synthesis Kit (1708890). Quantitative PCR (qPCR) was employed to amplify and detect target transcripts on the cDNAs, utilizing 5 μM primers. Detailed information about the specific primers used is provided in Supplemental Table 7.

The qPCR cycling conditions were set as follows: initial denaturation at 95°C for 20 seconds followed by 35 cycles of 95°C for 20 seconds and 60°C for 30 seconds, using the Fast SYBR Green Master Mix. Normalized CT values were used to quantify the expression of target transcripts through the ΔΔCT method. All qPCR runs included reverse transcription negative controls, which did not show amplification. Each qPCR experiment was conducted in both biological and technical triplicates.

### Immunofluorescence.

Cells were cultured as previously described and treated with ZNF638 siRNA for 6 hours. 48 hours after treatment, cells were seeded into 2-chambered slides at a density of 2.5 × 10^6^ cells/cm^2^. The cells were then fixed with 4% paraformaldehyde (Thermo Fisher Scientific) for 20 minutes and washed with PBS. For membrane permeabilization, cells were incubated with 0.01% Triton X for 5 minutes, followed by PBS washes and blocking with 10% normal goat serum. Primary antibodies (diluted 1:200) were applied overnight, then washed with PBS. Subsequently, slides were incubated with fluorescent-tagged goat anti-rabbit and goat anti-mouse secondary antibodies in 2% normal goat serum. Detailed information about the specific antibodies used is provided in Supplemental Table 6. For control, control + α-PDL-1, shRNA-ZNF638, and shRNA-ZNF638 + αPDL-1 samples, slides were deparaffinized and rehydrated using xylene and graded ethanol (100%, 95%, 70%). Antigen retrieval was performed by steaming slides in citrate buffer for 20 minutes. Permeabilization, blocking, and primary/secondary antibody incubations were conducted as described above. Images were captured using the EVOS M700 microscope. Quantification was done using 50 cells per treatment condition in triplicates using ImageJ.

### RNA-seq.

Ribosomal depleted RNA was extracted from patient-derived neurosphere cell lysate and sequenced using a lncRNA prep (250–300 bp, paired-end, 50 million reads). RNA-seq was performed by Novogene using the Illumina NovaSeq 6000 PE150 platform. FastQ files were prepared and aligned to the human Hg38 reference genome and subjected to external quality control. A custom bioinformatics pipeline from TE-Transcripts (M. Hammel Laboratory, Cold Spring Harbor Laboratory) was used to annotate retrotransposon counts (20). Differential expression analysis was conducted using DESeq2 with multiple testing corrections. Functional pathway and gene ontology analysis for each biological condition was conducted using “clusterProfiler” from Bioconductor. All plots were visualized in R using “ggplot” or Python using “seaborn.”

### ATAC-seq.

The preparation and sequencing of libraries for ATAC-seq were performed by Novogene. Paired-end sequencing was performed using the Illumina NovaSeq 6000 PE150 platform. FastQ files were aligned to the human Hg38 reference genome. Differentially accessible peaks were identified after DESeq2 normalization. Integrative Genomics Viewer was used to visualize tracks (version 2.17.4, Broad Institute) (53). Functional pathway and gene ontology analysis for each biological condition was conducted using “clusterProfiler” from Bioconductor.

### Proliferation assay.

shRNA-NC or shRNA-ZNF638 GBM cells were seeded at 1,000 cells/well in a 96-well E-plate in biological and technical triplicates. Cell proliferation was measured with xCELLigence RTCA DP instrument according to the manufacturer’s instructions (ACEA Bioscience) and visualized for over 7 days in culture or until cell proliferation plateaued.

### Mitochondrial RNA isolation.

A172 cells were transfected with ZNF638 smart pool siRNA (Dharmacon L-013715-02-0050). After transfection, cytosolic RNAs were isolated using the subcellular protein fractionation kit (Thermo Fisher Scientific) as described by manufacturer’s instructions. Cytosolic RNAs were isolated using TRIzol LS (Ambion) at 3:1 to the cytosolic fraction. For RT-qPCR, cDNA synthesis was performed using Bio-Rad kit per manufacturer instructions and RT-qPCR were performed using mtDNA-specific primers (Supplemental data), the data were normalized to ACTB mRNA levels.

### Intracranial orthotopic xenografts.

The mouse GBM cell line (SB-28) cell line was transduced with either lentivirus CTL shRNA and ZNF638 shRNA with an mCherry tag were cultured for 72 hours and sorted using BD FACS Aria (BD Biosciences). The sorted mCherry-positive cells were maintained in cell culture for 3 days. The harvested cells were used for implantation. 2 × 10^4^ cells resuspended in 3 μL PBS and implanted into the right frontal lobe of immunocompetent 6-week-old C57BL/6 female mice were obtained from The Jackson Laboratory using the following coordinates (anterior-posterior (AP), 1.5 mm; dorsal-ventral (DV), 3 mm; medial-lateral (ML), 2 mm). After tumor establishment on day 7, mice were treated with an anti PD-L1 (Bio cell) via i.p. injection for every 3 days until the death of the mice. Tumor volumes were measured and calculated twice per week using the modified ellipsoid formula 1/2 × (length × width^2^). Brain tumors were harvested immediately after sacrifice, fixed with 4% paraformaldehyde, and sectioned for histopathology. Whole blood was collected and allowed to clot at room temperature, then the serum was isolated by centrifuging at 1,000–2,000*g* for 10 minutes. The resulting supernatant and tumor tissues were preserved for downstream analysis, including ELISA and qPCR. All animal experiments were approved by the Institutional Animal Care and Use Committee at the University of Miami and performed in accordance with the guidelines.

### Immune profiling.

For immune profiling, the tumor tissue was digested using previously described methods from Newton et al., 2018 (54). In brief, tumors were removed from adjacent brain using microdissection. Tumor tissues were mechanically dissociated on a 40 μm strainer and washed with PBS before transferring into 96-well round-bottom plates (Thermo Fisher Scientific). Samples were stained with 1:1,000-diluted LIVE/DEAD Fixable Stains (BioLegend) in PBS for 10 minutes on ice. Following a wash step, cells were resuspended in FcR Blocking Reagent (Miltenyi Biotec) at a 1:25 dilution in PBS/2% BSA (Sigma-Aldrich) for 10 minutes on ice. Fluorophore-conjugated antibodies diluted 1:50 were added to suspensions, and cells were further incubated for 20 minutes on ice. A list of antibodies can be found in Supplemental Table 6. Samples were acquired with Cytek Aurora (Cytek Biosciences) and analyzed using FlowJo (v10.7.2, BD Biosciences).

### Cytokine array panel.

Tumor samples were excised from sacrificed mice and homogenized in PBS supplemented with protease inhibitor and Triton-X. Proteins were isolated and cytokines were measured using Mouse Cytokine Array Panel (ARY006, R&D Biosystems). Membranes were imaged using ECL chemiluminescence (R&D Biosystems) and data analysis was performed using HLImage++ 6.2 software.

### IHC.

The IHC detection kit was purchased from Abcam (ab64264). Experiments were performed according to the manufacturer’s instructions. Anti-H3K9Me3 (Cell Signaling D4W1U) and anti-CD8 (Cell Signaling D4W2Z) were diluted 1:200. Images were captured using EVOS M700 microscope at 10 × and 20 × magnification.

### Tissue microarray.

Unstained tissue microarray slides (core size 1.0 mm) were procured from Tissue Microarray (GL2082a), encompassing brain tumor and brain tissue samples, including 43 GBM and 10 normal cerebrum, with duplicate cores per case. The slides were processed using an IHC detection kit from Abcam (ab64264), following the manufacturer’s instructions, with the ZNF688 antibody at a 1:200 dilution. Immunostained slides were scanned using a High-throughput VS120 Olympus slide scanner with × 4–× 40 air lenses. The scanned images were then analyzed in a blinded fashion using Qupath image viewer software (v0.5.0-Mac-x64), with curated regions of interest outlined manually.

### RNA immunoprecipitation qPCR.

A172 cells treated with either scramble or ZNF628 siRNA (5.0 × 10^6^ cells) were harvested, and cytoplasmic fractions were extracted using the Nuclear Extract Kit (Active Motif; no. 40010) according to the manufacturer’s instructions. To isolate RNA, an equal volume of 70% ethanol was added to the cytoplasmic fractions, and RNA purification was performed using the TRIzol method followed by DNAse digestion. The total RNA was dissolved in 38 μL RNase-free water. A total 2 microliters of total RNA were used as input, and the remaining RNA was divided into 2 tubes.

For each RNA immunoprecipitation pulldown, 2 μg of J2 antibody (SCICONS; no. 10010200) and mouse control IgG2a (Abcam; no. ab18413) were conjugated to 20 μL protein G agarose (Sigma-Aldrich; no. 16-266) by rotating overnight at 4°C. To digest single-stranded RNA, 1 μL of RNase A (Sigma-Aldrich; no. R6513) was added to each tube and mixed with 1 mL IP buffer (50 mmol/L Tris-HCl [pH 7.4], 125 mmol/L NaCl, 1 mmol/L EDTA, 0.1% Triton X-100). The RNA samples were incubated with antibody-conjugated protein G agarose beads overnight at 4°C. Beads were washed 3 times with IP buffer and then incubated in 50 μL proteinase K digestion solution (1× TE, 100 mmol/L NaCl, 1% SDS, and 1 μL of 20 mg/mL Proteinase K solution [Thermo Fisher Scientific; no. AM2546]) for 20 minutes at 45°C to isolate RNA.

After centrifugation, 50 μL of the supernatant was added to 300 μL Buffer RLT Plus from the RNeasy Plus Mini Kit (Qiagen; no. 74106) to purify the RNA. The final product containing dsRNA was denatured for 5 minutes at 95°C, followed by reverse transcription using qScript cDNA SuperMix (iScript cDNA Synthesis Kit, 1708890). qRT-PCR was then performed using the primers listed in Supplemental Table 7 with the CFX96 Touch Real-Time PCR Detection System.

### Flow cytometry.

GBM43 neurospheres treated with scramble or ZNF638 siRNA were trypsinized and fixed using the CytoPerm/Fix kit (BD). The cells were then stained overnight at 4°C with mouse anti-dsRNA antibody (Scicons). Following staining, the cells were washed and resuspended in FACS buffer (1% FBS, 0.9% sodium azide in PBS). Flow cytometry analysis was performed using a BD LSRFortessa Cell Analyzer. Up to 50,000 cells were collected and gated to exclude nonviable cells.

### Coimmunoprecipitation.

For immunoprecipitation, the A172 scramble and ZNF638 siRNA cells were lysed by RIPA lysis buffer using an Immunoprecipitation kit (ab206996) containing protease inhibitors. Lysates were extracted and incubated with protein A/G agarose for preclearing, then immunoprecipitated with the primary antibody (ZNF638) overnight followed by incubation with protein A/G agarose for 2.5 hours. The precipitates were eluted and subjected to SDS gels for Co-IP assay using SETDB1, TASOR, and MPHOSPH8 antibodies.

### ELISA.

Serum samples were collected and processed for ELISA following manufacturer protocol using IFN-γ (MIF00-1, R&D Biosystems), TNF-α (MTA00B-, R&D Biosystems), and IFN-α (42120-1, R&D Biosystems). Absorbance in each well was then read at 450 nm using an Spectramax M5 automated reader (Molecular Devices), and evaluated using system-associated Softmax pro software v4.8.

### Multiplex immunofluorescence.

Paraffin-embedded tissue slides underwent deparaffinization and permeabilization through washes with xylene and ethanol. Antigen retrieval was performed using a 10 mM sodium citrate buffer (pH 6.0), heated for 2 minutes in an 800 W microwave (GE model PEM31DFWW) at full power. Following antigen unmasking, the slides were blocked using Background Buster (Innovex Biosciences, NB306) and FcR blocking solution (Innovex Biosciences, NB309).

Next, tissue sections were incubated at room temperature for 60 minutes with an 11-plex primary antibody cocktail, followed by multiple washes (3 times with dH2O). This was followed by secondary antibody incubation, with sections washed again (3 times with PBS and 3 times with dH2O). Subsequently, the tissue sections were counterstained with 1 μg/mL DAPI (Thermo Fisher Scientific) for pixel-pixel registration reference. Imaging was conducted using an Axio Imager.Z2 scanning fluorescence microscope (Carl Zeiss) equipped with a 20 ×, 0.8 NA Plan-Apochromat (Phase-2) nonimmersion objective (Carl Zeiss) and a 16-bit ORCA-Flash 4.0 sCMOS digital camera (Hamamatsu Photonics). Each labeling antibody was captured sequentially at its specific wavelength and digitized individually using ZEN2 imaging software (16-bit). Channels for antibodies of interest (dsRNA[green], PD-L1[yellow], CD8^+^[cyan], and DAPI[blue]) were compared between samples qualitatively. Patient characteristics and pathologies are described in Supplemental Table 5.

### Human GBM ICI response and survival analysis.

RNA count matrices and survival data were obtained from Zhao et. al 2019 (55) on a cohort of 38 patients treated with Anti-PD1 immunotherapy. Survival and response data on 3 cohorts of patients with Melanoma treated with anti-PD1 therapy or CTLA-4 blockade obtained from Van Allen et al. 2015, Snyder et al. 2015, and Hugo et al. 2017 (56–58). Integration methods are described by Noviello et al. 2023 (59). Boxplots for responders and nonresponders in both GBM and melanoma cohorts were generated in R using ggplot. Kaplan-Meier survival analysis was calculated and visualized in PRISM. Patients with low and high ZNF638 expression were stratified based on median expression value. Statistics are detailed below and in Supplemental Table 8.

### Statistics.

All experiments were conducted with biological replicates or triplicates and confirmed with technical triplicates. Data are shown as mean ± SEM. Full details for statistics are detailed within each subsection and within Supplemental Table 8. Tests used include unpaired 2-tailed *t* test, Mann-Whitney test, 1-way ANOVA, and log-rank (Kaplan-Meier) tests. *P* values of less than 0.05 were considered significant.

### Data availability.

All deidentified data will be made available from the corresponding author upon request and signature of data transfer agreement. Please see the attached Supporting Data Values file for access to raw data.

### Study approval.

All animal experiments were approved by the Institutional Animal Care and Use Committee at the University of Miami and performed in accordance with the guidelines (protocol #22-117-adm01).

## Author contributions

JC, DS, and AHS were involved in study initiation and design. JC, DS, JFD, CKR, JRC, AAM, SW, AJH, Vaidya Govindarajan, YZ, AMS, MJMV, DM, Vasundara Govindarajan, SRR, VML, MA, TMRN, ET, AA, RT, NS, CD, KJ, MIDLF, RAN, TMRN, MEI, RJK, AI, AN, JH, MC, KBC, MEF, DB, and AHS contributed to data collection, analysis, and running experiments. JC prepared the manuscript, which was reviewed and approved by all authors. AHS supervised all aspects of this work.

## Supplementary Material

Supplemental data

Unedited blot and gel images

Supporting data values

## Version 1 03/17/2025

Electronic publication
