# Supplementary material for: Activating antiviral immune responses potentiates immune checkpoint inhibition in glioblastoma models
Source: J Clin Invest. 2025 Mar 17;135(6):e183745. doi: 10.1172/JCI183745 (PMC11910234; doi:10.1172/JCI183745)
Supplement: Supplemental data [file jci-135-183745-s078.pdf]

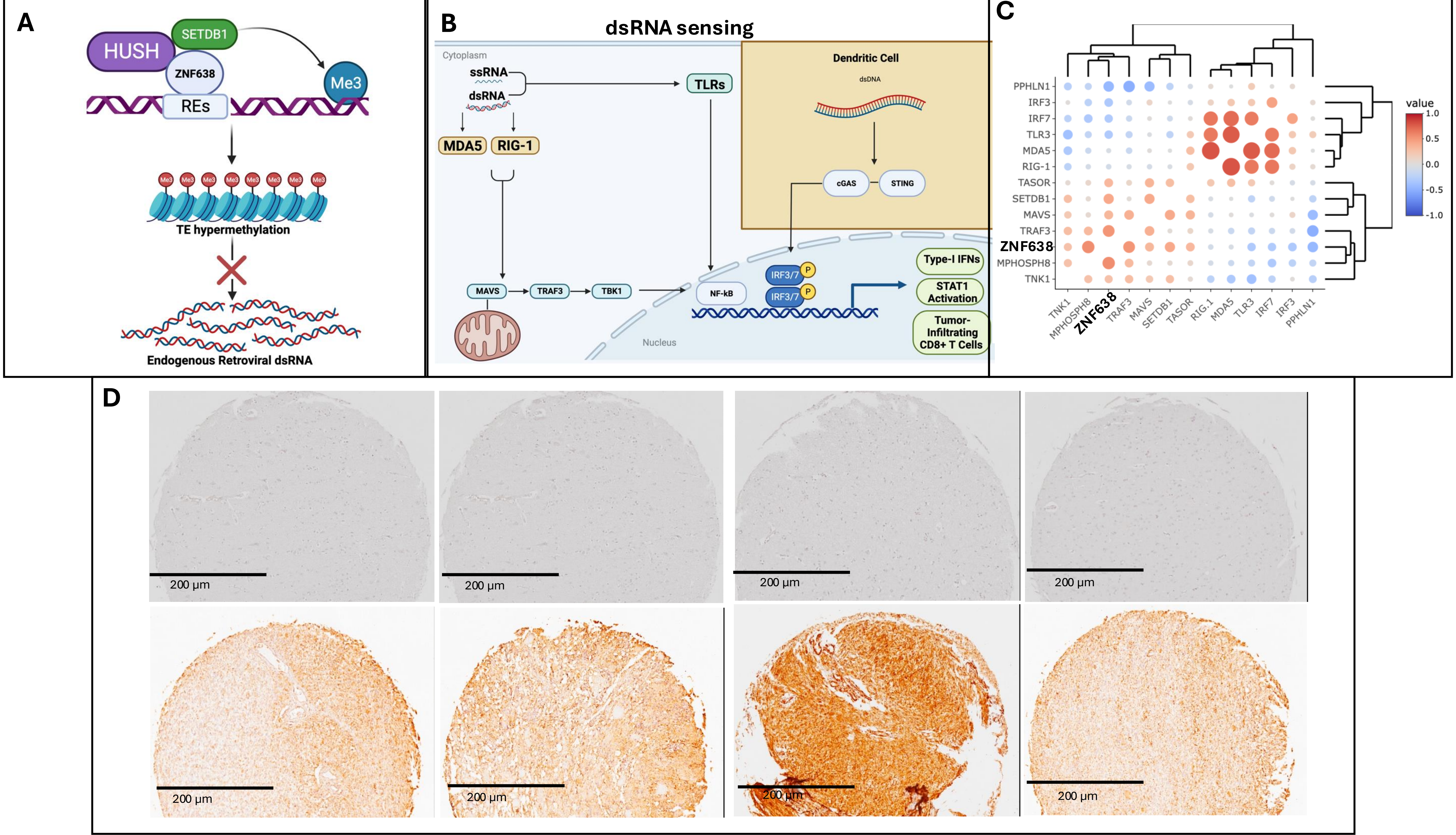

A
HUSH
SETDB1
ZNF638
Me3
REs
TE hypermethylation
Endogenous Retroviral dsRNA
B
dsRNA sensing
Cytoplasm
ssRNA
dsRNA
TLRs
MDA5
RIG-1
MAVS
TRAF3
TBK1
NF-kB
IRF3/7
P
Nucleus
Dendritic Cell
dsDNA
cGAS
STING
Type-I IFNs
STAT1 Activation
Tumor-Infiltrating CD8+ T Cells
C
PPHLN1
IRF3
IRF7
TLR3
MDA5
RIG-1
TASOR
SETDB1
MAVS
TRAF3
ZNF638
MPHOSPH8
TNK1
TNK1
MPHOSPH8
ZNF638
TRAF3
MAVS
SETDB1
TASOR
RIG.1
MDA5
TLR3
IRF7
IRF3
PPHLN1
value
1.0
0.5
0.0
-0.5
-1.0
D
200 µm
200 µm
200 µm
200 µm
200 µm
200 µm
200 µm
200 µm

Supplementary Data Figure 1. Role of ZNF638 in retroelement expression and immune signaling for gliomas A) Representation of ZNF638-mediated repression of retroelement dsRNA. Made with BioRender. B) Schematic of innate immune dsRNA sensing pathways to stimulate Type 1 IFN responses and downstream effectors. Made with BioRender. C) Correlation matrix using TCGA GBM (n=617) and LGG (n=516) bulk RNA sequencing datasets. ZNF638 and components of the retroviral silencing complex (SETDB1, TASOR, MPHOSPH8) are significantly positively correlated. ZNF638 is negatively correlated with dsRNA sensors (RIG-I, MDA5, TLR3) and downstream effectors (IRF3, IRF7). All circles shown have p-value <0.05. D) Representative images of immunohistochemistry against ZNF638. ZNF638 appears to be markedly enriched in GBM tissue samples (n=5) compared to matched normal cerebrum (n=5).

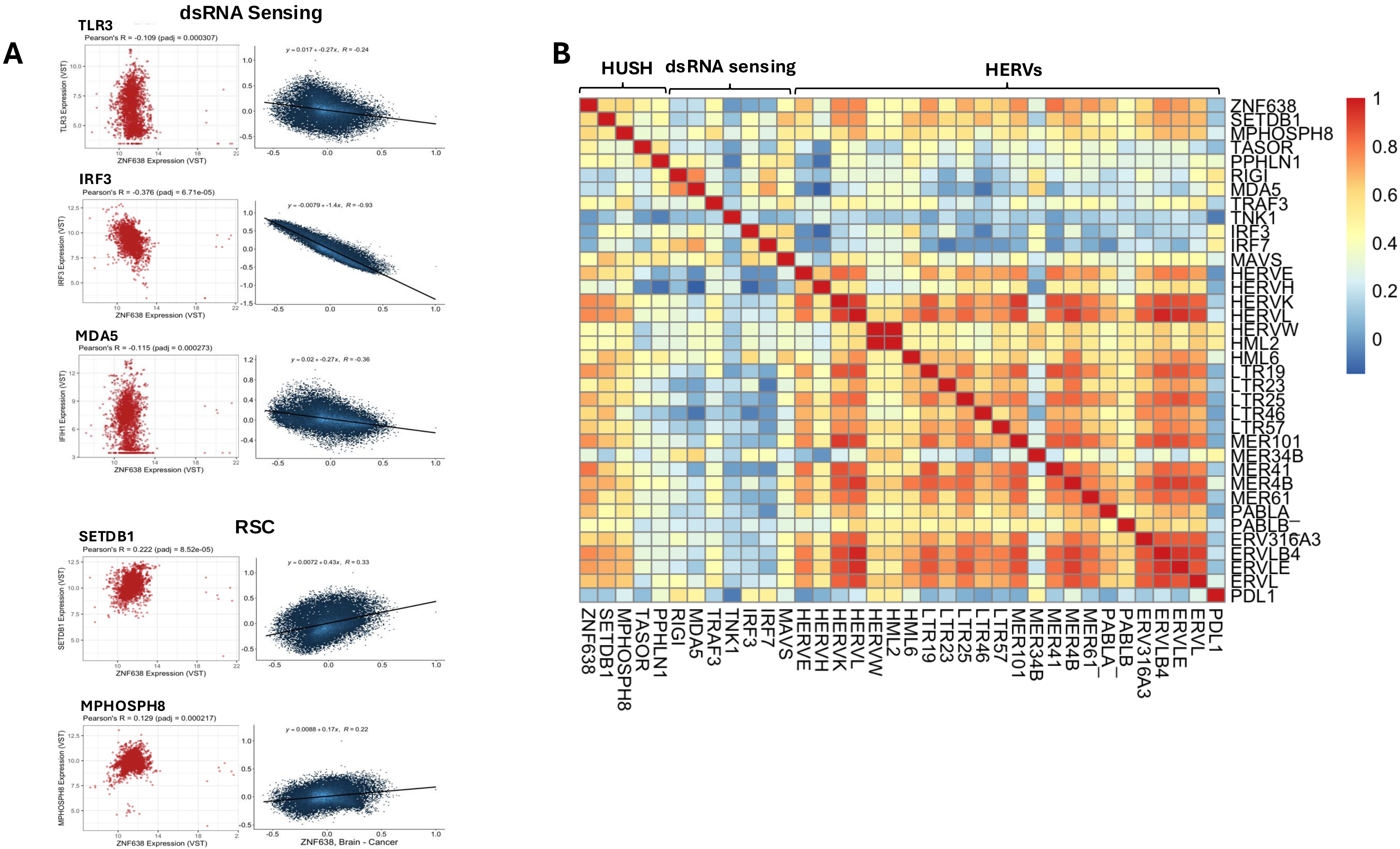

A
dsRNA Sensing
TLR3
Pearson's R = -0.109 (padj = 0.000307)
IRF3
Pearson's R = -0.376 (padj = 6.71e-05)
MDA5
Pearson's R = -0.115 (padj = 0.000273)
RSC
SETDB1
Pearson's R = 0.222 (padj = 8.52e-05)
MPHOSPH8
Pearson's R = 0.129 (padj = 0.000217)
ZNF638 Expression (VST)
ZNF638, Brain - Cancer
B
HUSH
dsRNA sensing
HERVs
ZNF638
SETDB1
MPHOSPH8
TASOR
PPHLN1
RIGI
MDA5
TRAF3
TNK1
IRF3
IRF7
MAVS
HERVE
HERVH
HERVK
HERVL
HERVW
HML2
HML6
LTR19
LTR23
LTR25
LTR46
LTR57
MER101
MER34B
MER41
MER4B
MER61_
PABLA_
PABLB_
ERV316A3
ERVLB4
ERVLE
ERVL
PDL1

Supplementary Data Figure 2. A) Transcriptomic analysis from patient GBM tumors reveals that ZNF638 is negatively correlated with dsRNA signaling and positively correlated with HUSH mediators SETDB1 and MPHOSPH8 (ARCHS4 database, n=1091 samples, RED=gene expression, BLUE=genome wide co-expression, $p<0.01$). B) Correlation matrix demonstrates enrichment of interferon-stimulated genes with expression of several REs and negative association between the HUSH complex and MDA5 signaling using the Reactome Pathways database. Gene ontology analysis demonstrates ZNF638 and HUSH complex are directly associated with increased inhibition of NK cell activation and Type 1 IFN signaling. TE = transposable elements.

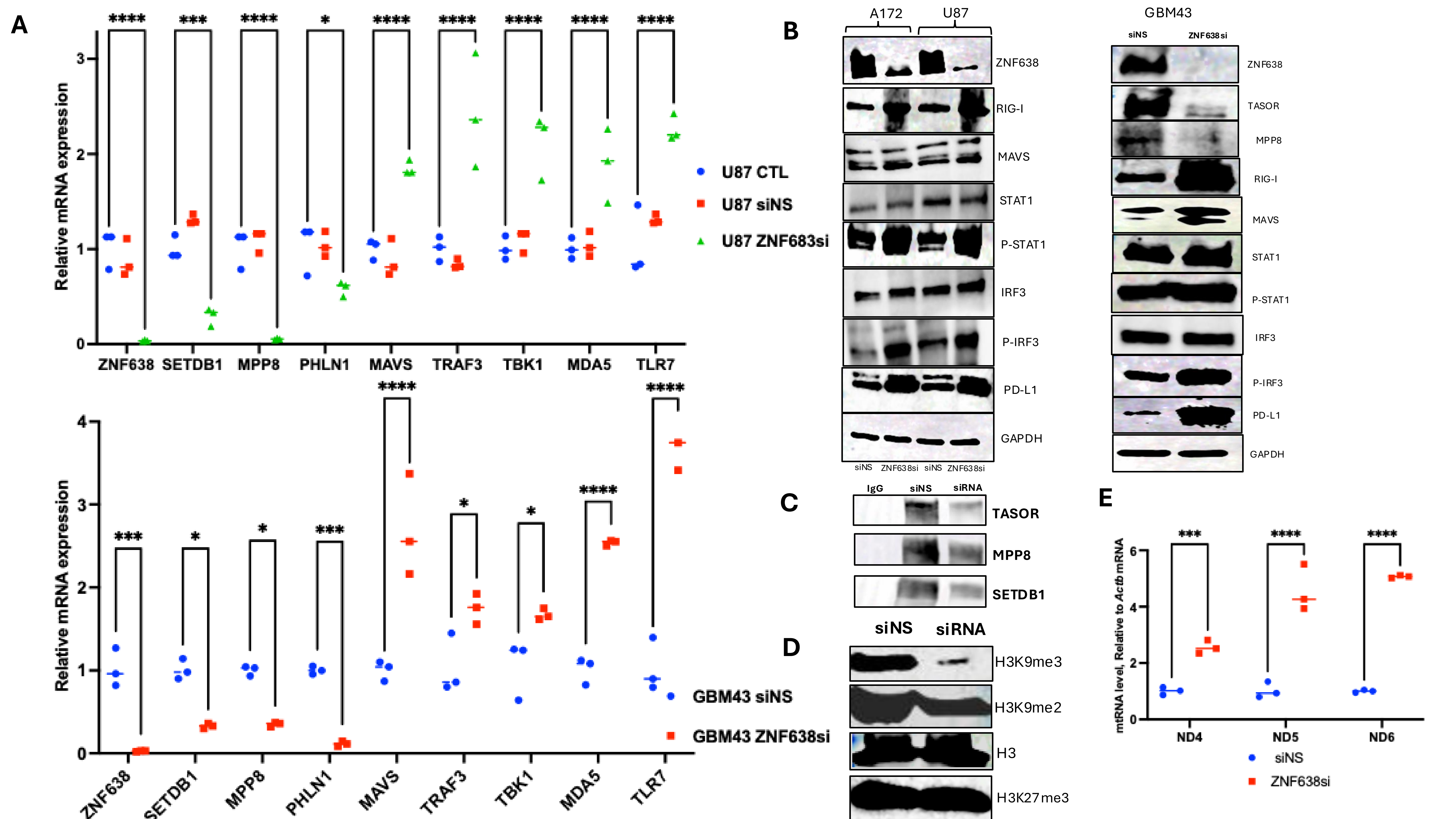
A
Relative mRNA expression
ZNF638
SETDB1
MPP8
PHLN1
MAVS
TRAF3
TBK1
MDA5
TLR7
U87 CTL
U87 siNS
U87 ZNF683si
GBM43 siNS
GBM43 ZNF638si
B
A172
U87
GBM43
siNS
ZNF638si
ZNF638
RIG-I
MAVS
STAT1
P-STAT1
IRF3
P-IRF3
PD-L1
GAPDH
TASOR
MPP8
C
IgG
siNS
siRNA
TASOR
MPP8
SETDB1
D
siNS
siRNA
H3K9me3
H3K9me2
H3
H3K27me3
E
mtRNA level, Relative to Actb mRNA
ND4
ND5
ND6
siNS
ZNF638si

Supplementary Data Figure 3. A) Knockdown of ZNF638 mRNA transcripts decreases expression of ZNF638, SETDB1, PPHLN1, and MPP8, and increased expression of MAVS, TRAF3, TBK1, MDA5, and TLR7 as measured by qPCR in both U87 and patient-derived GBM43. (ANOVA, ****P < 0.0001, ***P < 0.001, **P < 0.01 (right)). B) Western Blot confirms that ZNF638 KD in A72, U87, and GBM43 reduces expression of HUSH via MPHOSPH8 and TASOR and increases expression of RIG-I, MAVS, phospho-IRF3, phospho-STAT1, TLR3, and PD-L1 A172, U87, and GBM43 C) Co-immunoprecipitation with KD of ZNF638 in A172 results in loss of HUSH complex (TASOR, MPHOSPH8, and SETDB1). D) Knockdown of ZNF638 with siRNA results in loss of H3K9me3 in A172 based on Western Blot (performed in biological duplicate, ***p<0.001, *p<0.05). E) Knockdown of ZNF638 mRNA transcripts led to significantly increased mitochondrial dsRNA levels compared to control in A172 cells.

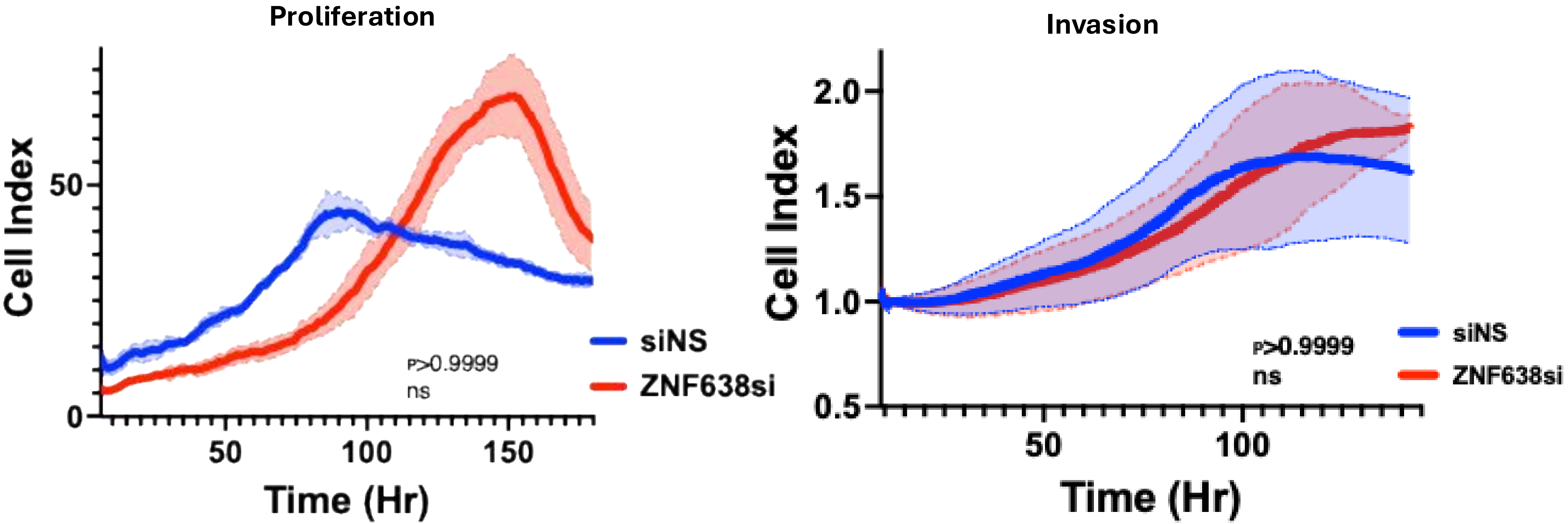
Proliferation
Cell Index
50
0
50
100
150
Time (Hr)
P>0.9999
ns
siNS
ZNF638si
Invasion
Cell Index
2.0
1.5
1.0
0.5
50
100
Time (Hr)
P>0.9999
ns
siNS
ZNF638si

Supplementary Data Figure 4. xCelligence proliferation assay and invasion assay demonstrate no significant difference in proliferation or invasion after ZNF638 KD in A172 compared to CTL.

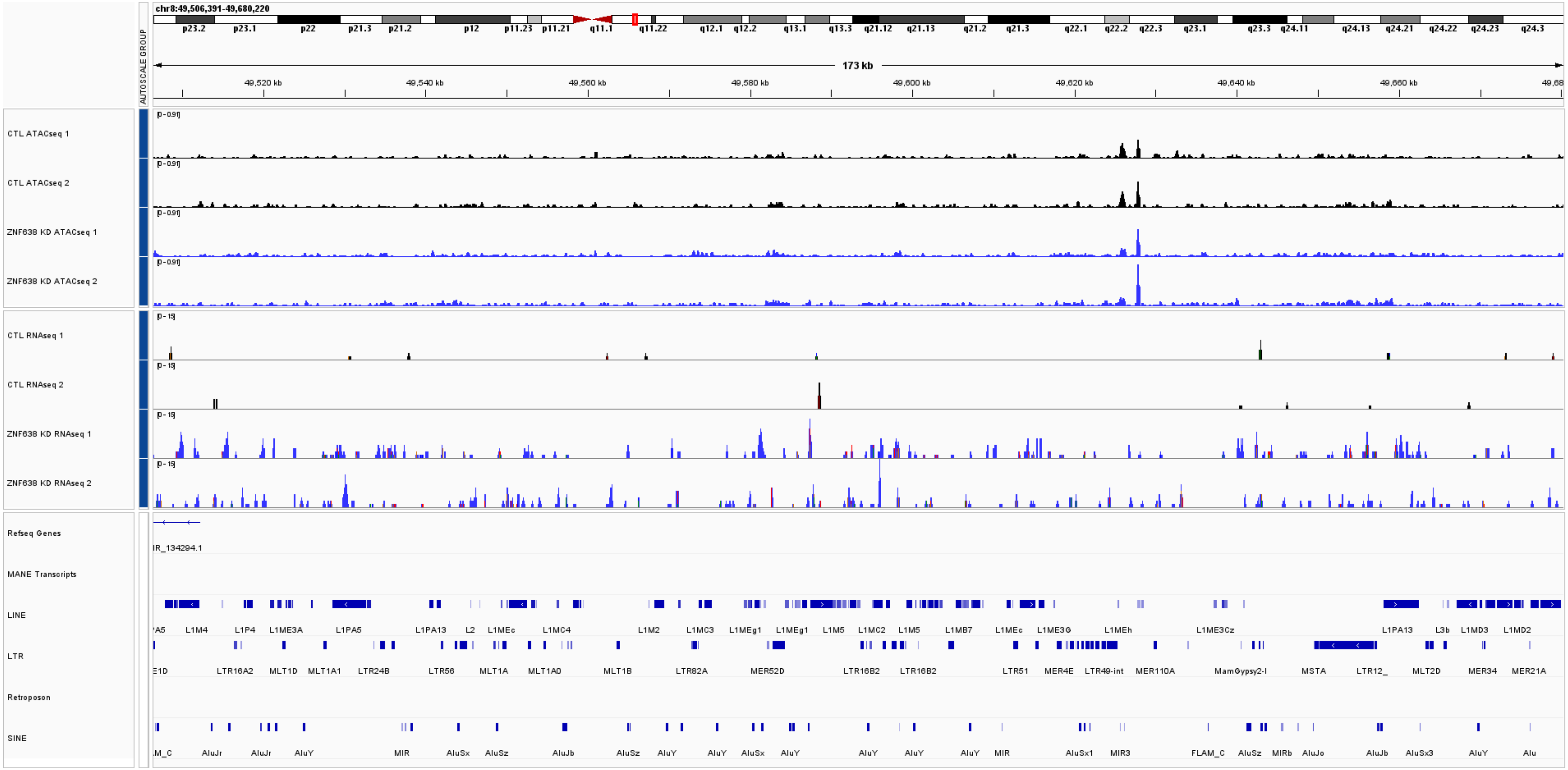

chr8:49,506,391-49,680,220
p23.2
p23.1
p22
p21.3
p21.2
p12
p11.23
p11.21
q11.1
q11.22
q12.1
q12.2
q13.1
q13.3
q21.12
q21.13
q21.2
q21.3
q22.1
q22.2
q22.3
q23.1
q23.3
q24.11
q24.13
q24.21
q24.22
q24.23
q24.3
AUTOSCALE GROUP
173 kb
49,520 kb
49,540 kb
49,560 kb
49,580 kb
49,600 kb
49,620 kb
49,640 kb
49,660 kb
CTL ATACseq 1
CTL ATACseq 2
ZNF638 KD ATACseq 1
ZNF638 KD ATACseq 2
CTL RNAseq 1
CTL RNAseq 2
ZNF638 KD RNAseq 1
ZNF638 KD RNAseq 2
Refseq Genes
MANE Transcripts
LINE
LTR
Retroposon
SINE
L1M4
L1P4
L1ME3A
L1PA5
L1PA13
L2
L1MEc
L1MC4
L1M2
L1MC3
L1MEg1
L1MEg1
L1M5
L1MC2
L1M5
L1MB7
L1MEc
L1ME3G
L1MEh
L1ME3Cz
L1PA13
L3b
L1MD3
L1MD2
LTR16A2
MLT1D
MLT1A1
LTR24B
LTR56
MLT1A
MLT1A0
MLT1B
LTR82A
MER52D
LTR16B2
LTR16B2
LTR51
MER4E
LTR49-int
MER110A
MamGypsy2-I
MSTA
LTR12_
MLT2D
MER34
MER21A
AluJr
AluJr
AluY
MIR
AluSx
AluSz
AluJb
AluSz
AluY
AluY
AluSx
AluY
AluY
AluY
AluY
MIR
AluSx1
MIR3
FLAM_C
AluSz
MIRb
AluJo
AluJb
AluSx3
AluY

Supplementary Data Figure 5. Intronic and retroelement transcripts significantly elevated upon ZNF638 knockdown on global scale. We have included a representative track of chromosome 8 to demonstrate globally elevated intronic retroelement expression by RNAseq. This does not appear to correlate with chromatin accessibility via ATACseq. Analyzed and made with Integrative Genomics Viewer (IGV). Intronic and repeat element transcripts annotated by RepeatMasker.[49]

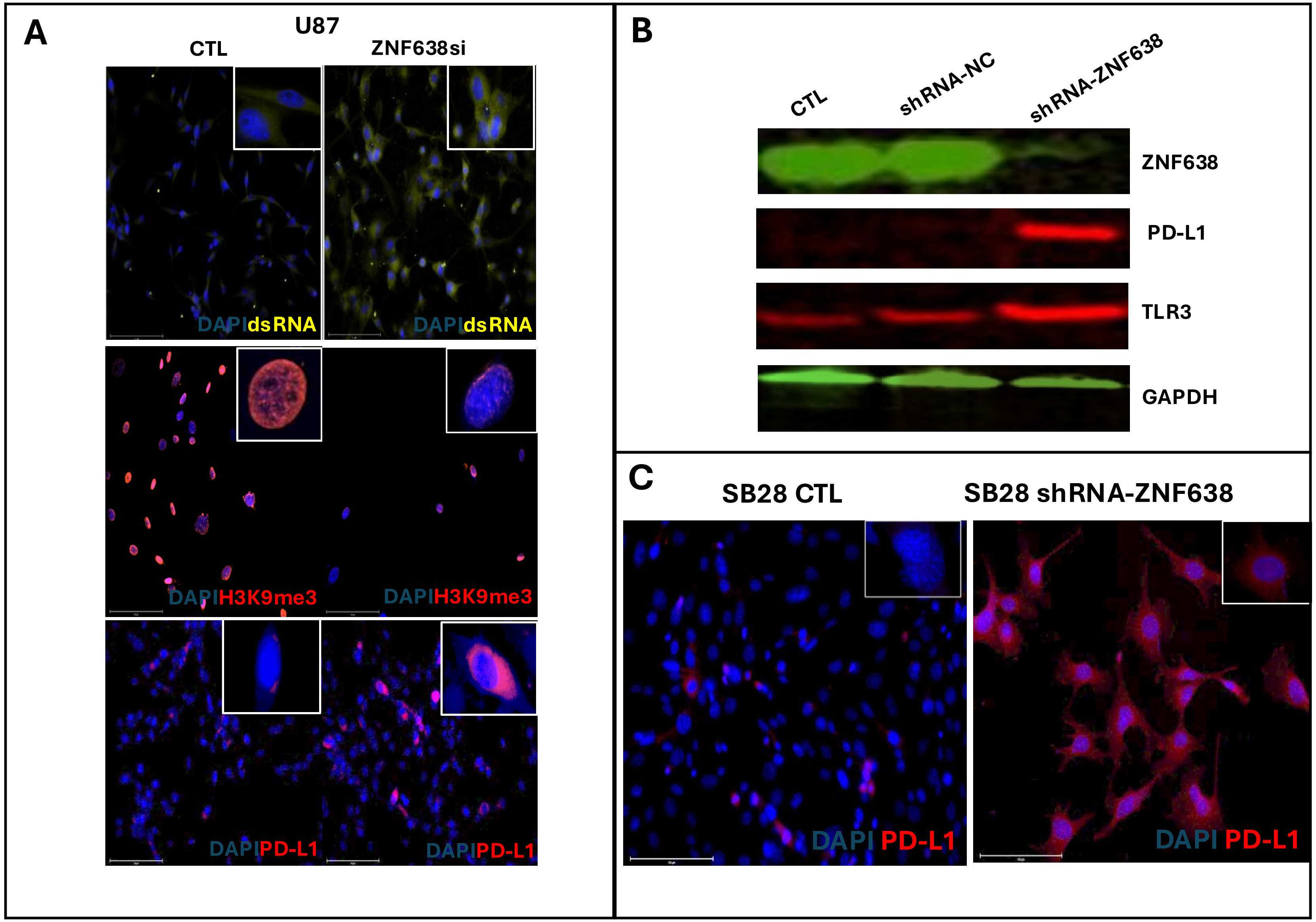
A
U87
CTL
ZNF638si
DAPI dsRNA
DAPI dsRNA
DAPI H3K9me3
DAPI H3K9me3
DAPI PD-L1
DAPI PD-L1
B
CTL
shRNA-NC
shRNA-ZNF638
ZNF638
PD-L1
TLR3
GAPDH
C
SB28 CTL
SB28 shRNA-ZNF638
DAPI PD-L1
DAPI PD-L1

Supplementary Data Figure 6. Immunostaining of ZNF638 KD in human and mouse glioma cell lines. A) ZNF638 KD in U87 results in increased dsRNA levels, PD-L1 expression, and histone 3 lysine 9 trimethylation (H3K9me3). DAPI (cell nuclei) – Blue, PD-L1 – Red, dsRNA – Yellow. B) Western blot showing increased protein expression of PD-L1 and TLR3 in a mouse glioma cell line (SB28) with ZNF638 KD compared to control shRNA. C) Immunofluorescence of SB28 cells demonstrates ZNF638 KD increases PD-L1 expression. DAPI (cell nuclei) – Blue, PD-L1 – Red.

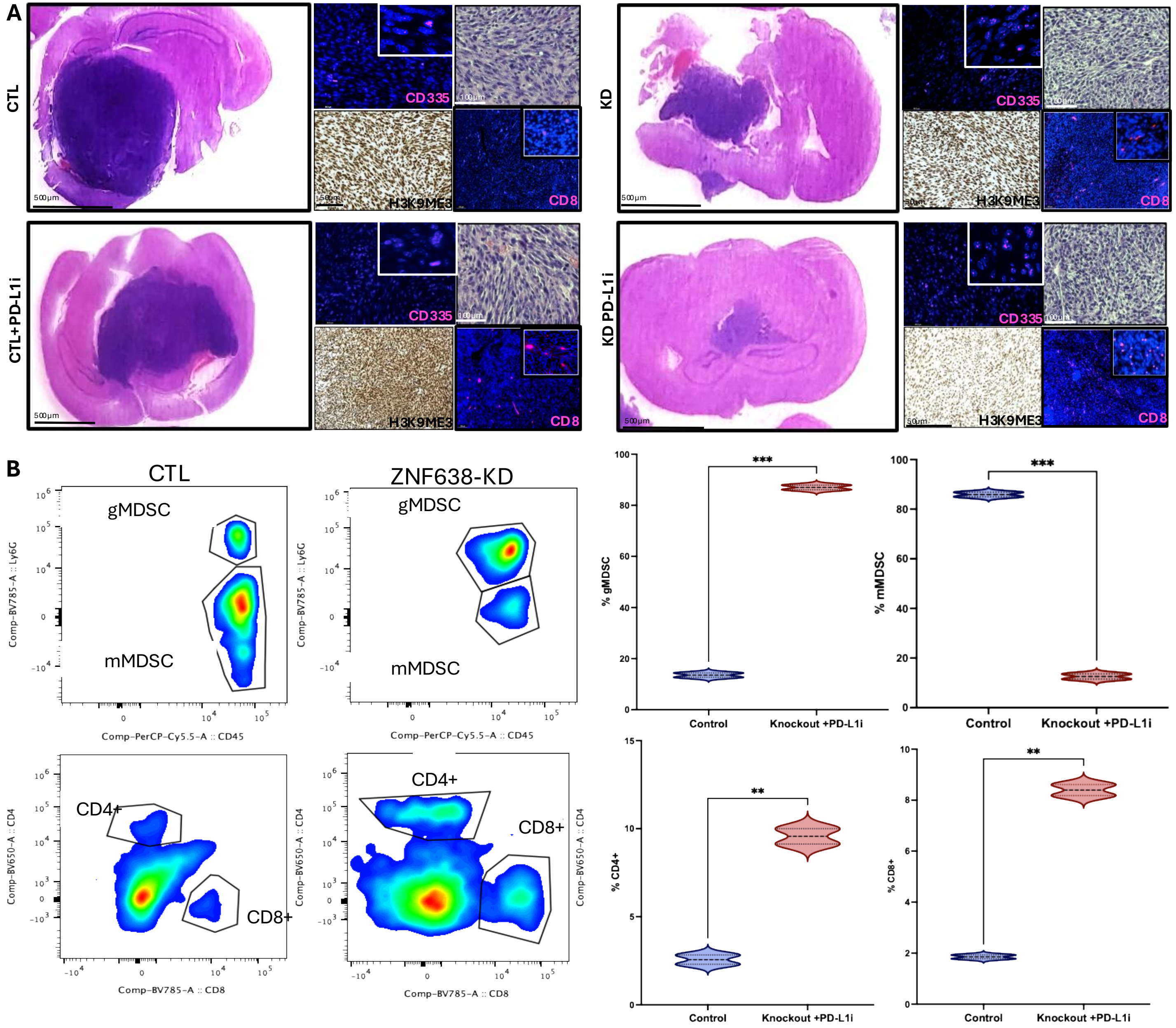

A
CTL
CD335
H3K9ME3
CD8
CTL+PD-L1i
KD
KD PD-L1i
B
CTL
ZNF638-KD
gMDSC
mMDSC
Comp-BV785-A :: Ly6G
Comp-PerCP-Cy5.5-A :: CD45
CD4+
CD8+
Comp-BV650-A :: CD4
Comp-BV785-A :: CD8
% gMDSC
% mMDSC
% CD4+
% CD8+
Control
Knockout +PD-L1i
***
**

Supplementary Data Figure 7. A) H&E and Immunofluorescence demonstrates increased tumor-infiltrating lymphocytes and reduced overall tumor size in ZNF638 KD + α PD-L1 mice. Immunofluorescence depicted upregulated CD8+ cell and CD335+ cell (Natural Killer cell) infiltration correlated with decreased global H3K9me3. B) KD of ZNF638 results in increased populations of CD8+, CD4, and granulocytic myeloid derived suppressor (gMDSC) cells and decreased populations of monocytic myeloid-derived suppressor cells (mMDSC).

Supplementary Table 1: Patient IHC

Patient	Pathology diagnosis
US23-26024	Glioblastoma IDH wild type
US23-23371	Glioblastoma IDH wild type
US23-23057	Glioblastoma IDH wild type
US23-22644	Glioblastoma IDH wild type
US23-22578	Glioblastoma IDH wild type
US23-22302	Glioblastoma IDH wild type
US23-22213	Glioblastoma IDH wild type
US23-5870	Diffuse glioma IDH1-mutant
US24-7724	Diffuse astrocytoma, IDH1 mutant
US24-2949	Unremarkable cerebral cortex and white matter
US24-6792	Unremarkable cerebral cortex and white matter
US23-20248	Cerebral grey and white matter with reactive gliosis

Supplementary Table 2: Tissue Microarray – Patient Characteristics

Patients Age	Sex	Pathology diagnosis
22	M	Glioblastoma
22	M	Glioblastoma
44	F	Glioblastoma
44	F	Glioblastoma
25	M	Glioblastoma

25	M	Glioblastoma
59	F	Glioblastoma
59	F	Glioblastoma
22	F	Glioblastoma
22	F	Glioblastoma
59	M	Glioblastoma
59	M	Glioblastoma
33	F	Glioblastoma
33	F	Glioblastoma
42	M	Glioblastoma
42	M	Glioblastoma
27	F	Glioblastoma
27	F	Glioblastoma
32	M	Glioblastoma
32	M	Glioblastoma
63	F	Glioblastoma
63	F	Glioblastoma
67	F	Glioblastoma
67	F	Glioblastoma
41	F	Glioblastoma
41	F	Glioblastoma
40	F	Glioblastoma
40	F	Glioblastoma
80	M	Glioblastoma
80	M	Glioblastoma
40	M	Glioblastoma
40	M	Glioblastoma

41	F	Glioblastoma
41	F	Glioblastoma
42	M	Glioblastoma
42	M	Glioblastoma
45	M	Glioblastoma
45	M	Glioblastoma
62	M	Glioblastoma
48	F	Glioblastoma
48	F	Glioblastoma
47	F	Glioblastoma
47	F	Glioblastoma
72	M	Cerebrum tissue
72	M	Cerebrum tissue
58	F	Cerebrum tissue
58	F	Cerebrum tissue
45	F	Cerebrum tissue
45	F	Cerebrum tissue
38	M	Cerebrum tissue
34	M	Cerebrum tissue
34	M	Cerebrum tissue
37	M	Cerebrum tissue

Supplementary Table 2. Summary of characteristics and pathologies of patients whose samples were visualized in tissue microarray. GBM = glioblastoma.

Supplementary Table 3
GBM43: ZNF638 KD vs. Sham Vector: Significantly Upregulated Pathways

Pathways	NES	p-adj
Negative Regulation of Viral Genome Replication	-1.71	TNIP1, SRPK2, N4BP1,APOBEC3G, ISG20
Positive Regulation of T-cell Activation	1.36	SMARCE1, HLA-DQA1, SPN, IL18, RASAL3
Positive Regulation of T-cell Proliferation	1.37	SPN, IL18, RASAL3, CD276, AIF1, NCKAP1L
Immune-response activating cell surface receptor signaling pathway	1.22	CACNB4, LCP2, CD79B, HCK, FCGR2B
Positive regulation of immune system process	1.22	SMARCE1, PPP2R3C, AQP3, HLA-DQA1, SPN
T-cell Proliferation	1.37	SPN, IL18, CTPS1, RASAL3, LGALS9C
I-kappa/NF-kappaB complex	1.32	BCL3
Cytokine-cytokine receptor interaction	1.75	1E-10
JAK-STAT signaling	1.67	1.087E-07
cAMP signaling	1.67	8.27E-10
Ras signaling	1.66	1.34E-09
cGMP-PKG signaling	1.64	1.85E-07
Pi3K-Akt signaling	1.60	1.68E-10
Kaposi sarcoma – associated herpesvirus infection	1.58	1.29E-06
MAPK signaling	1.56	2.04E-08

Pathways in cancer	1.55	1E-10
Coronavirus – COVID-19	1.55	2.79E-06
Human papillomavirus infection	1.53	1.62 E-08
Herpes simplex virus 1 infection	1.48	3.44E-09
Regulation of actin cytoskeleton	1.48	2.23E-05
Human cytomegalovirus infection	1.46	0.00011
Pathways of neurodegeneration	1.44	1.52E-07

Supplementary Table 3. Summary of cellular upregulated in patient-derived glioblastoma neurospheres based on RNA-sequencing and GSEA analysis.

Supplementary Table 4
GBM43: ZNF638 KD vs. Sham Vector: Significantly Upregulated Retrotransposons

Retrotransposon	Enrichment P Value	Log_2FoldChange
LTR12C	0.019	1.58
L1MB4	0.044	1.33
L1MD1	0.041	1.29
L2A	0.025	1.29
L1M4	0.047	1.29
Tigger1	0.008	1.26

Supplementary Table 4. Summary of retrotransposons upregulated in patient-derived glioblastoma neurospheres based on RNA-sequencing analysis.

Supplementary Table 5

Patient	Age	Sex	Pathology	Location
1	60	M	rGBM	Temporal
2	54	M	rGBM	Frontal
3	51	M	nGBM	Frontal

Supplementary Table 5. Summary of characteristics and pathologies of patients whose samples were analyzed with Multiplex Immunofluorescence. rGBM= recurrent glioblastoma, nGBM= newly diagnosed glioblastoma.

Supplementary Table 6. Primary Antibodies

Target	Host	Use	Manufacturer/product number	Dilution
ZNF638	Rabbit	WB, IP	Thermo scientific PA5 37001	1:1000
MPHOSPH8	Rabbit	WB, IP	SIGMA HPA040035	1:1000
RIG1	Rabbit	WB, IF	ABCAM AB180675	1:1000
MAVS	Mouse	WB	Santa Cruz sc-166583	1:1000
TLR3	Mouse	WB	ABCAM AB13915	1:1000
PDL1	Mouse	WB, IF	BioXCell BP0101	1:1000

H3k9Me3	Rabbit	IHC, IF	Invitrogen 49-1008	1:200
GAPDH	Rabbit	WB	Millapore SigmaG9545	1:1000
CD3	Mouse	Flow	Biolegend 100237	1:300
CD4	Mouse	Flow	Biolegend 100469	1:300
CD8A	Mouse	Flow	Biolegend 100749	1:300
CD69	Mouse	Flow	Biolegend 1004511	1:300
Ly6C	Mouse	Flow	Biolegend 128035	1:300
CD11c	Mouse	Flow	Biolegend 117339	1:300
Ly6G	Mouse	Flow	Biolegend 127645	1:300
P2RY12	Mouse	Flow	Biolegend 848003	1:300
CD45R	Mouse	Flow	Biolegend 103211	1:300
CD279	Mouse	Flow	Biolegend 135217	1:300
IA/IE	Mouse	Flow	Biolegend 107629	1:300
CD11b	Mouse	Flow	Biolegend 101211	1:300
F4/80	Mouse	Flow	Biolegend 123129	1:300
CD68	Mouse	Flow	Biolegend 137025	1:300
SETDB1	Rabbit	WB, IP	Preoteintech 11231-1-AP	1:1000
TASOR	Rabbit	WB, IP	Cell signaling 92278	1:1000
Anti-Histone H3	Rabbit	ChIP Grade	Abcam ab1791	1:1000
Di-Methyl-Histone H3 (Lys9) (D85B4) XP®	Rabbit	ChIP Grade	Abcam ab4658	1:1000
Anti-Histone H3 (tri methyl K9)	Rabbit	ChIP Grade	Abcam ab8898	1:1000
Anti-Histone H3 (tri methyl K27)	Mouse	ChIP Grade	Abcam ab6002	1:1000
PD-L1	Rabbit	WB	Cell signaling 13684	1:1000

IGg	Mouse	ChIP Grade	Abcam ab18413	1:1000
J2	Mouse	Flowcytometry	SCICONS 10010200	1:200
CD335	Rabbit	IF	Invitrogen PA5-102860	1:200

Supplementary Table 7 Primers

Target	Species	Primers 5'-3'
ZNF638	Human	F: ATGTCGAGACCCAGGTTAATCC
		R: TGTGGCCCCATGTTCTGATAA
SETDB1	Human	F: AGGAACTTCGGCATTTCATCG
		R: TGTCCCGGTATTGTAGTCCCA
MPP8	Human	F: GATGCTACTACCACACTCCGTG
		R: TAAGCAGCCTGAAGACCGTTGG
PHLN1	Human	F: GAGACGATCATTCTGCAAGCA
		R: TCTCTCGCATAATGGGAAGAGTA
MAVS	Human	F: CAGGCCGAGCCTATCATCTG
		R: GGGCTTTGAGCTAGTTGGCA
TRAF3	Human	F: TCACGGAGGTGATTAGAATGACT
		R: ACAACCACGTCTATGGCCTTT
TBK1	Human	F: GGCGGCTAGAAGAGGCTTTG
		R: CTCCGTCAGCTCGGTGTAG
GAPDH	Human	F: TGTGGGCATCAATGGATTTGG
		R: ACACCATGTATTCCGGGTCAAT
ZNF638	Mouse	F: CAAAGGCCACGAGCACCTAAT
		R: TCTTGGAAGACCCATAGAGCC

RIG1	Mouse	F: TTGAGGACTTGACGAATCTGC
		R: CTTGTTGTGCTGCAAAAAGAGAG
TLR3	Mouse	F: GTGAGATACAACGTAGCTGACTG
		R: TCCTGCATCCAAGATAGCAAGT
NFKB	Mouse	F: CTGGGCACCAGTTCGATGG
		R: GACAGCATAAGGCACACACTT
IL-6	Mouse	F: GGCGGATCGGATGTTGTGAT
		R: GGACCCCAGACAATCGGTTG
TNF-Alpha	Mouse	F: GGAACACGTCGTGGGATAATG
		R: GGCAGACTTTGGATGCTTCTT
MERVs	Mouse	F: AACTCGTTCCCAGAACACTCC
		R: AGCGGGGTAGGGAAAGTACAA
GAPDH	Mouse	F: AATGGATTTGGACGCATTGGT
		R: TTTGCACTGGTACGTGTTGAT
ERV3-1	Human	F: AGGAAGAGGGAGTATGCGGAAAG
		R: CAAGTCTGAACTGGGATGTGAGC
ERVW-1	Human	F: GCAATACTACATACACAACCAACTCC
		R: GGCACTAAGAATGAGAGGAAGCAC
L1ORF1	Human	F: AGGAAAGCCCATCAGACTAACAGT
		R: GGCCTGGTGGTGACAAAATCT
L1ORF2	Human	F: TCATAAAGCAAGTCCTCAGTGACC
		R: GGGGTGGAGAGTTCTGTAGATGTC
ALU	Human	F: CAACATAGTGAAACCCCGTCTC
		R: GCCTCAGCCTCCCGAGTAG
L1HS	Human	F: GGTTACCCTCAAAGGAAAGCC
		R: GCCTGGTGGTGACAAAATCTC
TLR7	Human	F: TCCTTGGGGCTAGATGGTTTC

		R: TCCACGATCACATGGTTAGCCTTTG
HERV-K GAG	Human	F: AGGAGGTCAGGTGCCTGTAACATT
		R: TGGTGCCGTAGGATTAAGTCTCCT
HERV-K POL	Human	F: TCACATGGAAACAGGCAAAA
		R: AGGTACATGCGTGACATCCA
HERV-K ENV	Human	F: CTGCCAAACCTGAGGAAGAA
		R: GCAGTCCAAAATTGGTTGGT

Supplementary Table 8: Statistics

Sample	Figure	Statistical Test	Notes
Correlation Matrix and Plots	1C/1D	Pearson correlation coefficient and t-test	P<0.05 for all depicted correlations
Immunohistochemistry	4A	Un-paired t-test	53978 (n=43) vs. 9.29 (n=10), p<0.0001
CPTAC	4D	Un-paired t-test	n=12 vs. n=99
GBM43 Western Blot Quantification	4E	Un-paired t-test	P<0.0001, technical triplicates
A172 qPCR	4F	Two-way ANOVA	P=<0.0001, n=3, technical triplicates
J2 IP-qPCR	5B	One-way ANOVA	ANOVA, p<0.0001, technical triplicates

A172 pIRF3 Immunofluorescence	5D	One-way ANOVA	ANOVA, $p<0.0001$,technical triplicates
Histone western blot quantification	5E	Nested t test	$P=0.0321$ technical triplicates
Probability of survival and tumor volume	8B-C	Curve comparison and One-way ANOVA	P=<0.0001, n=5 per group
SB28 cell lines- qPCR	8D	One-way ANOVA	$P=0.0004$, n=3, biological triplicates
ELISA (IFN alpha, TNF alpha and IFN gamma)	8E	One-way ANOVA	$p<0.001$, biological triplicates
ICI Response	9B	Mann-Whitney	$P_{GBM}=0.0034$, $P_{melanoma}=0.035$
ICI Survival	9C	Mantel-Cox	$P < 0.0001$
U87 and GBM43 qPCR	S3A	Unpaired t-test	P=<0.0001, technical triplicates
A172 Mitochondrial RNA	S3E	Unpaired t-test	$P<0.0001$, technical triplicates
Flow Cytometry	S7B	Unpaired t-test	$P_{gMDSC}=0.0003$, $P_{mMDSC}=0.0004$, $P_{CD4+}=0.0053$, $P_{CD8+}=0.0012$
