# Supplementary material for: Activating antiviral immune responses potentiates immune checkpoint inhibition in glioblastoma models
Source: J Clin Invest. 2025 Mar 17;135(6):e183745. doi: 10.1172/JCI183745 (PMC11910234; doi:10.1172/JCI183745)
Supplement: Unedited blot and gel images [file jci-135-183745-s079.pdf]

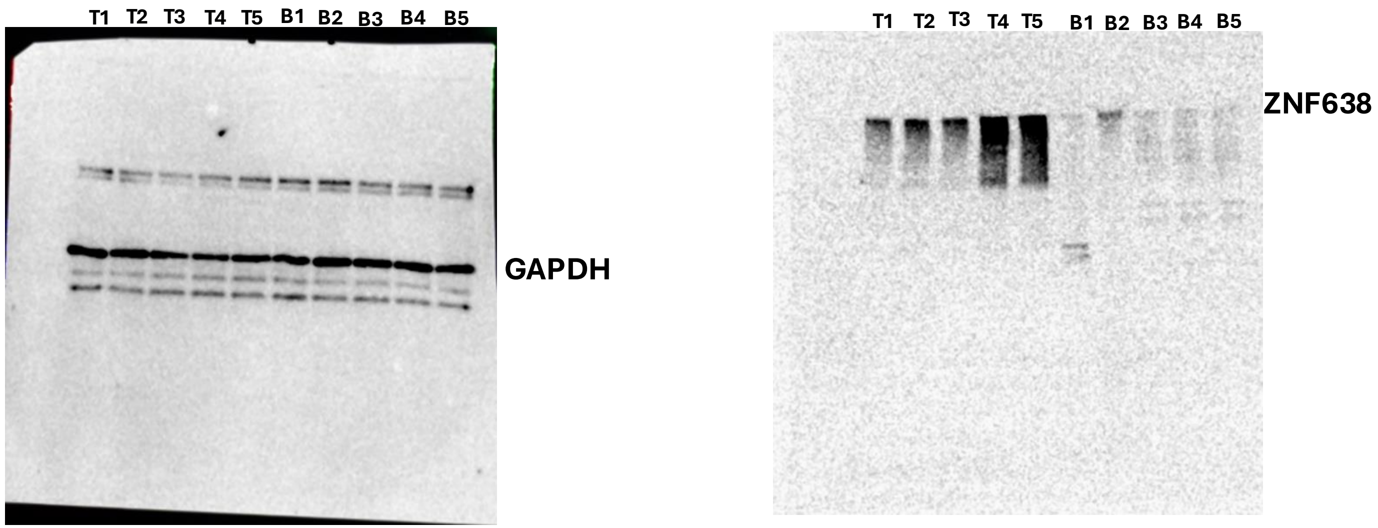
T1 T2 T3 T4 T5 B1 B2 B3 B4 B5
GAPDH
T1 T2 T3 T4 T5 B1 B2 B3 B4 B5
ZNF638

Figure 4C

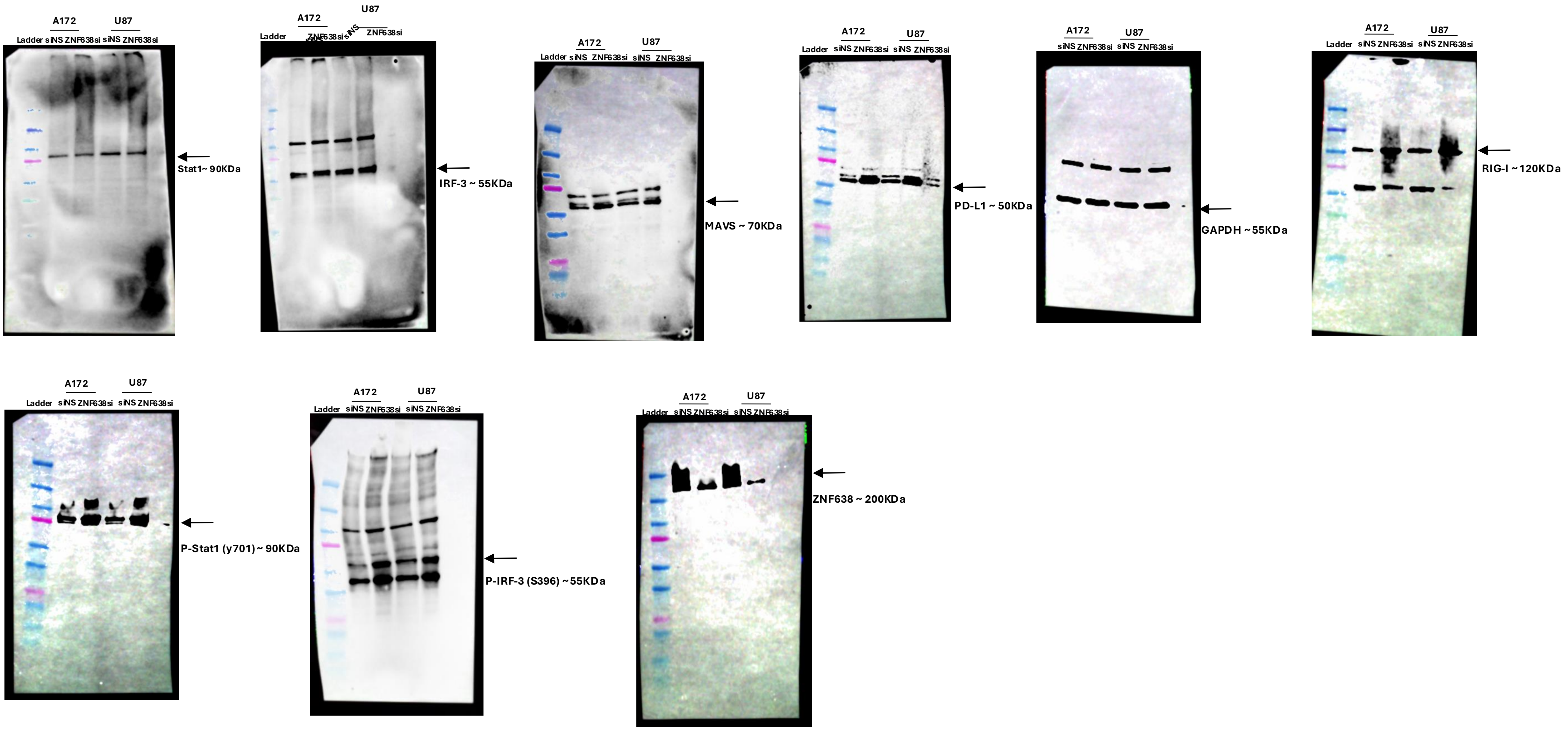

Figure S3A

A172 and U87 uncropped blots

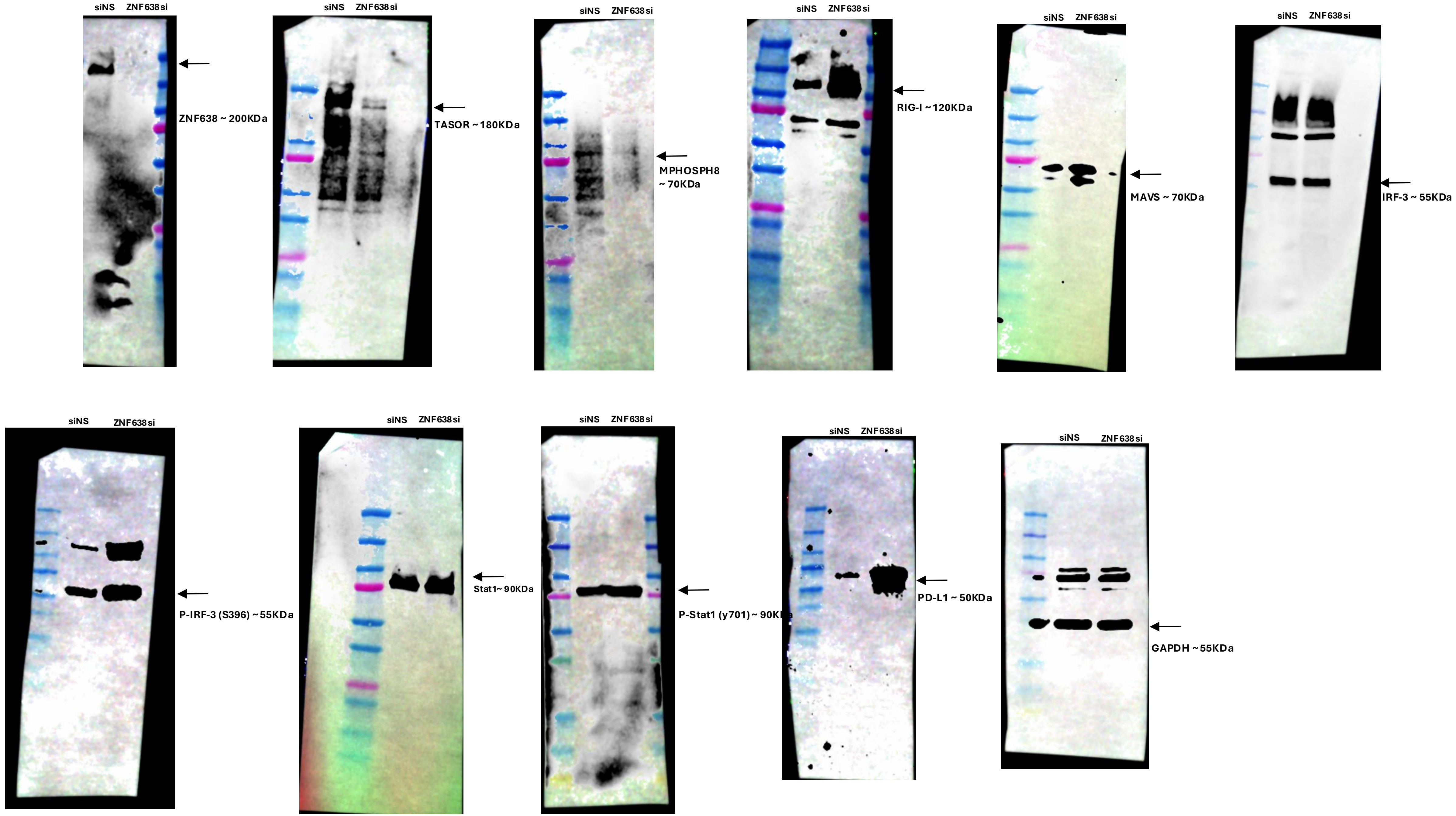

Figure S3A

GBM43 uncropped blots

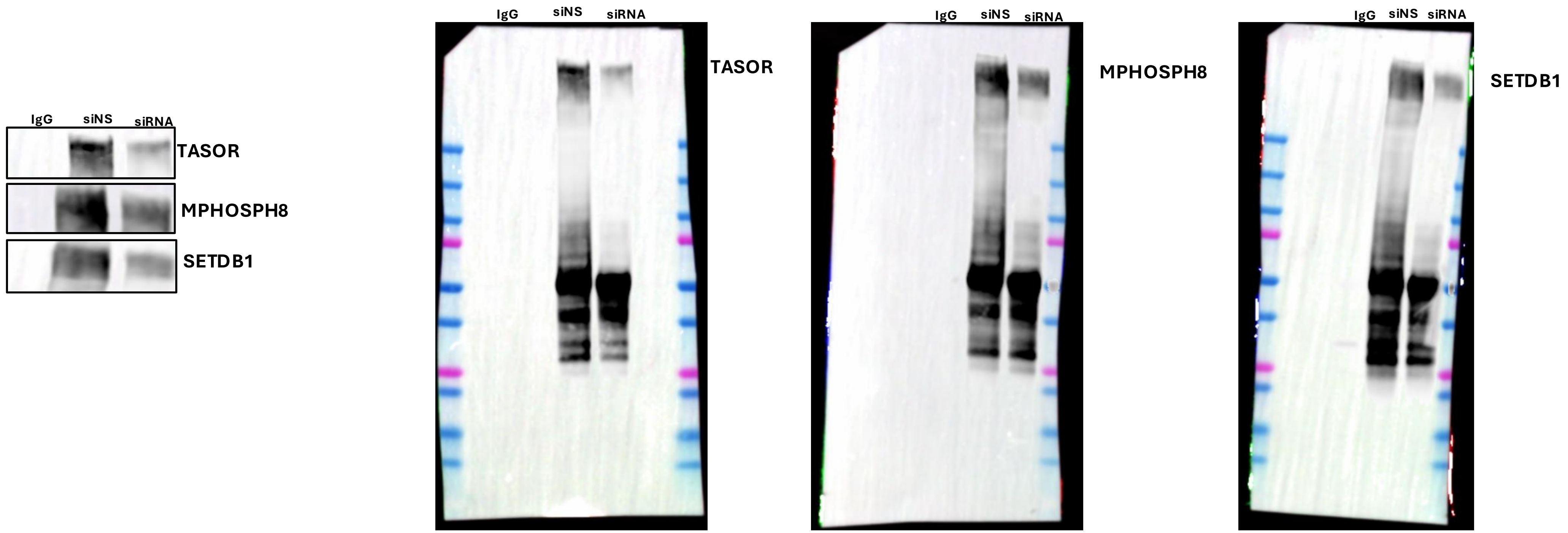
IgG
siNS
siRNA
TASOR
MPHOSPH8
SETDB1
IgG
siNS
siRNA
TASOR
IgG
siNS
siRNA
MPHOSPH8
IgG
siNS
siRNA
SETDB1

Figure S3C

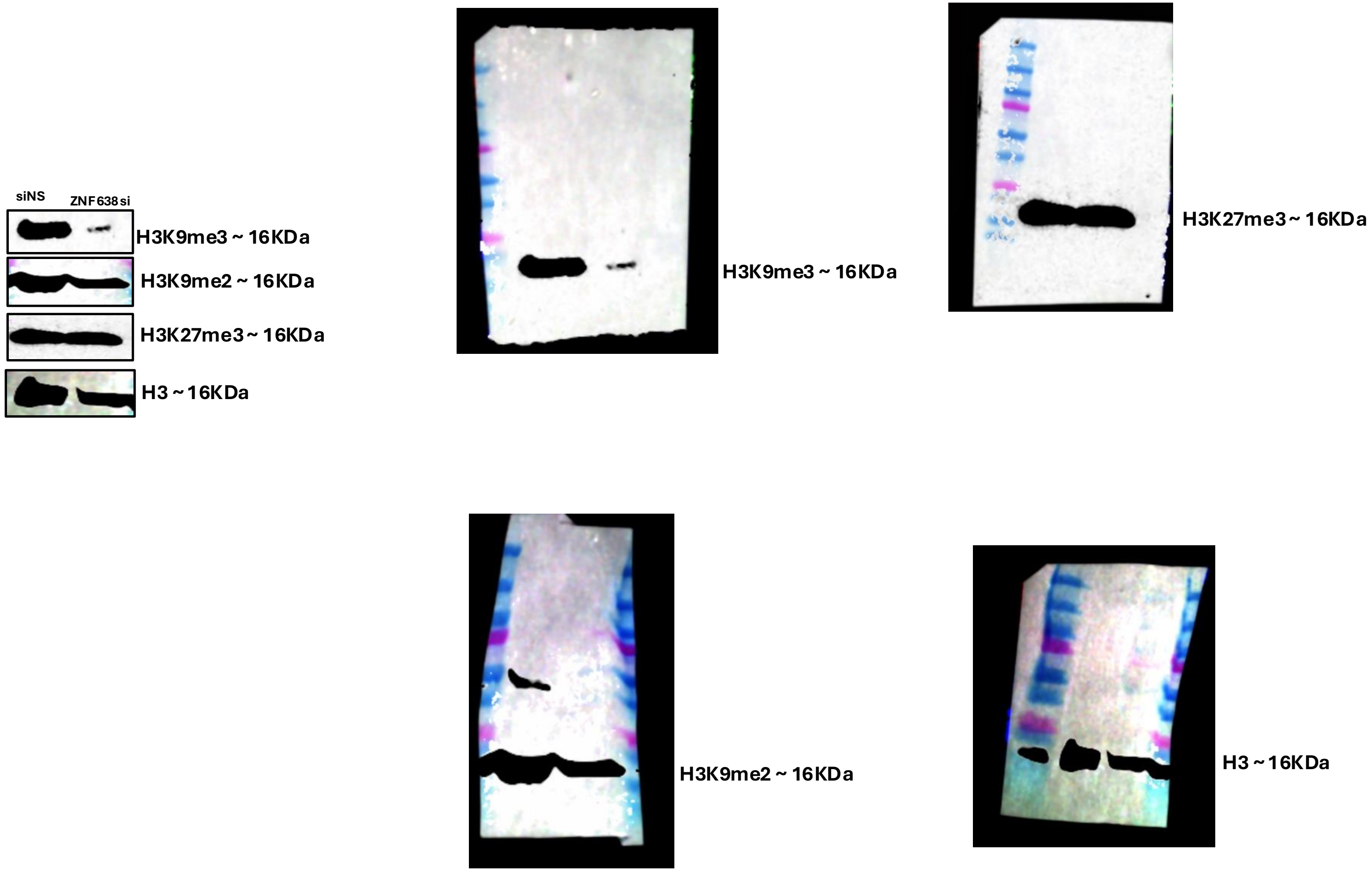
siNS
ZNF638 si
H3K9me3 ~ 16KDa
H3K9me2 ~ 16KDa
H3K27me3 ~ 16KDa
H3 ~ 16KDa
H3K9me3 ~ 16KDa
H3K27me3 ~ 16KDa
H3K9me2 ~ 16KDa
H3 ~ 16KDa

Figure S3D

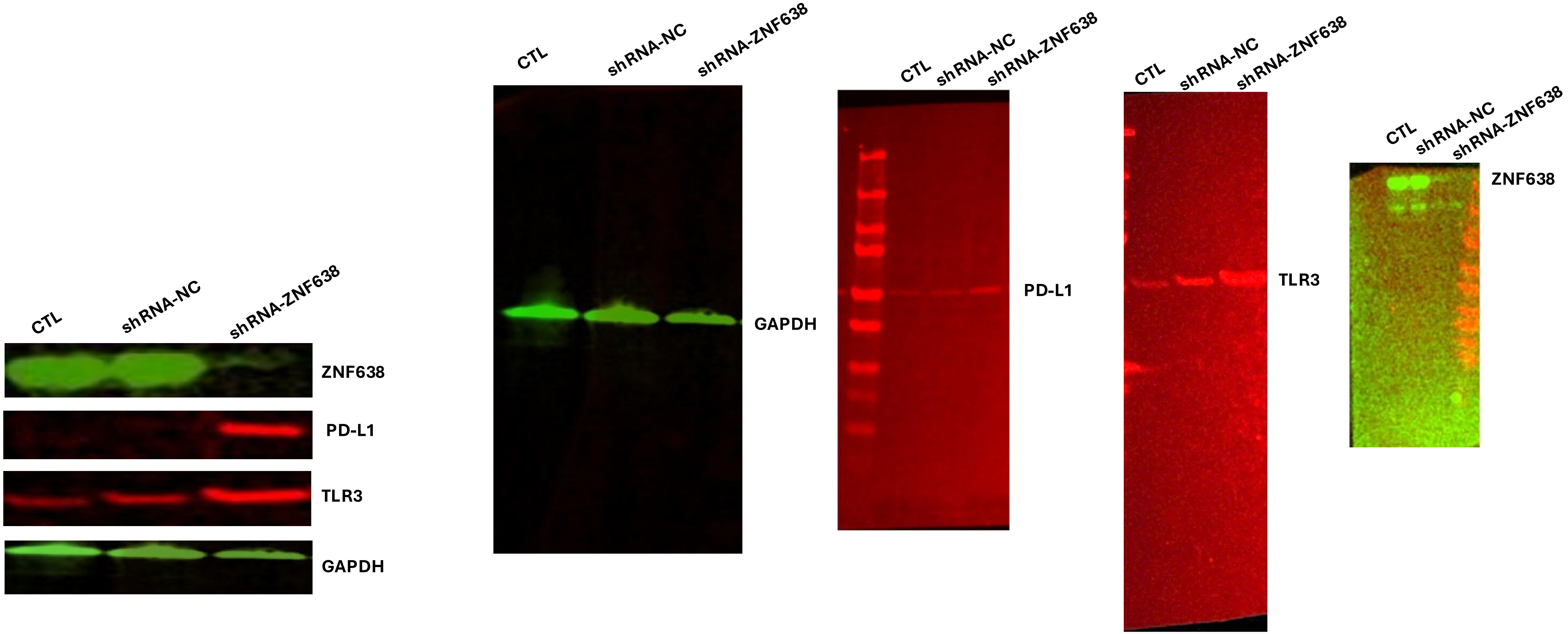
CTL
shRNA-NC
shRNA-ZNF638
ZNF638
PD-L1
TLR3
GAPDH
CTL
shRNA-NC
shRNA-ZNF638
GAPDH
CTL
shRNA-NC
shRNA-ZNF638
PD-L1
CTL
shRNA-NC
shRNA-ZNF638
TLR3
CTL
shRNA-NC
shRNA-ZNF638
ZNF638

Figure S6B
